# Impact of Secretion-Active Osteoblast-Specific Factor 2 in Promoting Progression and Metastasis of Head and Neck Cancer

**DOI:** 10.3390/cancers14092337

**Published:** 2022-05-09

**Authors:** Désirée Gül, Andrea Schweitzer, Aya Khamis, Shirley K. Knauer, Guo-Bin Ding, Laura Freudelsperger, Ioannis Karampinis, Sebastian Strieth, Jan Hagemann, Roland H. Stauber

**Affiliations:** 1 Department of Otorhinolaryngology, Head and Neck Surgery, Molecular and Cellular Oncology, University Medical Center, 55131 Mainz, Germany; andrea.schweitzer@gmx.de (A.S.); ayakhamis@uni-mainz.de (A.K.); laura.freudelsperger@unimedizin-mainz.de (L.F.); jan.hagemann@unimedizin-mainz.de (J.H.); 2 Oral Pathology Department, Faculty of Dentistry, Alexandria University, El Azareta, Alexandria, Egypt; 3 Institute for Molecular Biology, Centre for Medical Biotechnology (ZMB), University Duisburg-Essen, Universitätsstraße, 45117 Essen, Germany; shirley.knauer@uni-due.de; 4 Institute of Biotechnology, The Key Laboratory of Chemical Biology and Molecular Engineering of Ministry of Education, Shanxi University, Taiyuan 030006, China; dinggb2012@sxu.edu.cn; 5 Academic Thoracic Center, University Medical Center Mainz, Johannes Gutenberg University Mainz, 55131 Mainz, Germany; ioannis.karampinis@unimedizin-mainz.de; 6 Department of Otorhinolaryngology, University Medical Center Bonn, 53127 Bonn, Germany; sebastian.strieth@ukbonn.de

**Keywords:** metastases, HPV, biomarker, therapy resistance, methylation, oral cancer, protein secretion

## Abstract

**Simple Summary:**

Head and neck cancers (HNC) exhibit poor survival due to metastases. Our study identified osteoblast-specific factor 2 (OSF-2) as overexpressed in primary tumors, lymph node metastases, and the tumor microenvironment. High OSF-2 levels correlate with metastatic disease and reduced survival of HPV-negative HNC patients. OSF-2’s active secretion signal seems to promote metastases by supporting the tumor microenvironment via the ß1 integrin-induced PI3K and Akt/PKB signaling pathway. We suggest OSF-2 as a potential biomarker and drug target to control (HPV-negative) HNC metastasis and disease management.

**Abstract:**

Treatment success of head and neck cancer (HNC) is still hampered by tumor relapse due to metastases. Our study aimed to identify biomarkers by exploiting transcriptomics profiles of patient-matched metastases, primary tumors, and normal tissue mucosa as well as the *TCGA* HNC cohort data sets. Analyses identified osteoblast-specific factor 2 (OSF-2) as significantly overexpressed in lymph node metastases and primary tumors compared to normal tissue. High OSF-2 levels correlate with metastatic disease and reduced overall survival of predominantly HPV-negative HNC patients. No significant correlation was observed with tumor localization or therapy response. These findings were supported by the fact that OSF-2 expression was not elevated in cisplatin-resistant HNC cell lines. OSF-2 was strongly expressed in tumor-associated fibroblasts, suggesting a tumor microenvironment-promoting function. Molecular cloning and expression studies of OSF-2 variants from patients identified an evolutionary conserved bona fide protein secretion signal (^1^MIPFLPMFSLLLLLIVNPINA^21^). OSF-2 enhanced cell migration and cellular survival under stress conditions, which could be mimicked by the extracellular administration of recombinant protein. Here, OSF-2 executes its functions via ß1 integrin, resulting in the phosphorylation of PI3K and activation of the Akt/PKB signaling pathway. Collectively, we suggest OSF-2 as a potential prognostic biomarker and drug target, promoting metastases by supporting the tumor microenvironment and lymph node metastases survival rather than by enhancing primary tumor proliferation or therapy resistance.

## 1. Introduction

Head and neck cancers (HNC) are among the most common malignant neoplasms in humans [1,2]. The most common entity of HNC is solid squamous cell carcinoma (HNSCC) which is the sixth most malignant tumor worldwide with 890,000 new cases yearly and 450,000 deaths in 2018 [3]. Major risk factors associated with the development of HNC are tobacco use, alcohol consumption, and high-risk human papillomavirus infections (HPV) [2]. HNC occurs in 50% of patients with locoregional invasion and lymph node metastases [4]. Along with the late disease presentation, lack of suitable biomarkers, and corresponding drugs for individually targeted therapy approaches this is the reason that survival rates for HNC have not improved significantly within the last years [5,6,7]. In cases of developed distant metastases, HNC patients have a 5-year survival rate of less than 20% [8]. Furthermore, it is presumed that the presence of micrometastases leads to enhanced mortality and morbidity. A huge number of cancer deaths are caused by the haematogenous spread of cancer cells into distant organs in combination with metastases development [9,10]. Metastatic spread was often described as a late process in malignant progression, but recent work about breast cancer progression suggested that the dissemination of primary cancer cells to distant sites seems to be an early event [11,12]. Thus, the early formation of micrometastases might also play an important role in HNC tumorigenesis and progression, which need to be understood on the molecular and clinical levels.

The main prognostic parameters of head and neck squamous cell carcinoma are the location and size of the tumor, the presence of distant metastases, and the presence of cervical lymph node metastases [13]. However, since the prognosis according to the TNM classification is not sufficient to evaluate the disease outcome [14], there is a need to identify molecular biomarkers with prognostic and diagnostic relevance. Microarray gene expression studies suggested gene expression signatures associated with recurrent disease in HNC [15], or genes with diagnostic or prognostic potential [1,7,16,17,18].

A variety of factors, genetic as well as epigenetic, have been associated with the complex process of (HNC) metastasis. For example, among the list of frequently altered genes by methylation in HNC are also proteins responsible for cellular adhesion, migration, and extracellular matrix degradation such as APC, CDH1, SPP1, and TIMP3 [19,20]. We refer the interested reader to some detailed reviews [11,21,22,23,24,25]. Enhancing tumor metastasis can be influenced by increasing (local) tumor cell proliferation or/and by increasing the cell’s capability to migrate and survive in distant ‘hostile’ microenvironments. Here, a variety of proteases, anti-apoptotic proteins, and matricellular proteins, such as osteopontin have been suggested to impact metastasis [26,27,28,29,30]. However, in most cases, such proteins are not solely involved in metastasis but also impact tumorigenesis.

Regarding the tumor microenvironment, so called ‘niche proteins’ regulate various functions in the tumor-associated bed [31,32,33,34,35]. Cancer cells detach from the primary solid tumor and intravasate into the peripheral blood system to form new metastatic sites. Moreover, tumor cells in the bone marrow seem to form an important reservoir of cancer cells from which they also may re-circulate into other distant organs [36]. 

In this study, we identified OSF-2 up-regulation in a series of human HNC tumors and their corresponding metastases and normal tissue. The 93 kDa glycoprotein OSF-2 was originally identified as an adhesion molecule involved in bone formation, regulating differentiation of osteoblasts, and preferentially expressed in periosteum in bone tissues [37,38]. The OSF-2 protein was described to be composed of an N-terminal EMI domain (EMILIN family domain, cysteine-rich), a tandem repeat of four fas1 domains allowing binding of integrins, and glycans [39], and a carboxyl-terminal domain that includes a heparin-binding site [40]. Due to structure and sequence homologies, OSF-2 was classified as a member of the fasciclin 1 family [38]. In addition to OSF-2, the group of FAS1-similar proteins contains also βigh3 [41], stabilin I and II [42], and periostin-like-factor (PLF) [43]. However, the molecular functions of the predicted domains are not fully understood. In addition, OSF-2 was also found to be under- or overexpressed in various human cancers and other diseases [44,45,46,47]. Despite its history, the tumor-promoting or tumor-suppressing functional relevance of OSF-2 in cancer development and metastasis in the quite heterogeneous field of solid and liquid malignancies is still controversially discussed.

Here, our bioinformatic results regarding *OSF-2* up-regulation in HNC were independently verified on the mRNA and protein levels. To investigate the source of OSF-2 production, we isolated human HNC cancer cells and tumor-associated fibroblasts and found OSF-2 expression significantly upregulated in tumor-associated fibroblasts. We further analyzed OSF-2 isoform expression signatures in tumors, their corresponding lymph node metastases, and normal tissue as well as expression signatures in tumor cells and corresponding tumor-associated fibroblasts. We identified OSF-2 as a secreted protein, crucial for cell migration and survival under stress conditions via ß1 integrin-induced PI3K and Akt/PKB signaling pathway, classifying OSF-2 as a ‘tumor niche’ protein’ in HNC.

## 2. Materials and Methods

### 2.1. Antibodies, Chemicals, and Reagents

Ab used: α-OSF-2 (Biovendor, Heidelberg, Germany), α-GAPDH (sc-47724; Santa Cruz Biotechnology, Heidelberg, Germany), α-GFP (sc-8334; Santa Cruz Biotechnology, Heidelberg, Germany) α-Lamin B1 (NEB, Darmstadt, Germany), anti-Actin (A2066; Sigma Aldrich, Munich, Germany), anti-Vimentin (V9; Fisher Scientific, Schwerte, Germany), anti-p-Akt (9271; NEB, Darmstadt, Germany), anti-γH2AX (A300-081A, Bethyl Laboratories, Montgomery, TX, USA). Appropriate HRP-, Cy3- or FITC-conjugated secondary antibodies (Sigma Aldrich, Munich, Germany; Santa Cruz Biotechnology, Heidelberg, Germany) were used. Recombinant OSF-2 (RD1720450, sequence in Supplementary Table S5) was purchased from BioVendor, Heidelberg, Germany. PI3K-inhibitor (LY294002, Cat. No. 9901) was purchased from Cell Signalling Technology, Danvers, MA, USA. Reagents, such as cisplatin were from Sigma (Sigma Aldrich, Munich, Germany) or MSC (MSC UG&CoKG, Mainz, Germany) unless stated otherwise.

### 2.2. Cell Culture and OSF-2 Secretion

Authenticated and characterized cell lines (Fadu, SU8686, 1624, HOB18, Pancl, Carey24, MG63, HeLa, HEK293T were purchased from the *ATCC* repository, expanded, stocks prepared at early passages, and frozen stocks kept in liquid nitrogen. Thawed cells were routinely monitored by visual inspection and growth-curve analyses to keep track of cell-doubling times, and were used for a maximum of 20 passages for all experiments. Depending on the passage number from purchase, cell line authentication was further performed at reasonable intervals by short tandem repeat (STR) profiling. The Pica cell line was initially established from laryngeal squamous cell carcinoma as described by Siemer et al. [48]. The HNSCCUM-02T squamous cell carcinoma cell line was established by Welkoborsky et al. [49] and maintained as described before [50]. To avoid contamination Hela and HEK293T cells are cultivated and maintained physically separately. For primary tumor cell and CAF isolation, ethical approval has been obtained for the collection and use of HNC tumor biopsies. Primary tumor cells and CAFs were isolated from an independent patient cohort. For the isolation of primary cancer cells or CAFs, specimens were cut into pieces and enzymatically digested with collagenase type I/hyaluronidase (Sigma Aldrich, Munich, Germany) in RPMI-1640 (Invitrogen, Karlsruhe, Germany) at 37 °C overnight. Following digestion, dissociated cells were passed through a cell strainer, and epithelial cells were isolated by MACS^®^separation using CD326 (EpCAM) MicroBeads (Miltenyi Biotec GmbH, Bergisch Gladbach, Germany) according to the manufacturer’s recommendations. Cells were propagated for one week as [1] and subjected to analysis.

For the detection of secreted OSF-2 protein, HEK293T cells were grown in a normal medium until 70% confluency. Twelve hours after OSF-2-transfection cells were washed and the medium was replaced by 1% FCS containing medium for 24 h. The medium was harvested and analyzed by immunoblot.

To prepare the conditioned medium, HEK293T cells were transfected with OSF-2, incubated for 12 h, and the medium was exchanged containing 1% FCS. After 24 h incubation, the conditioned medium was collected, either used directly or stored at −80 °C.

### 2.3. Microarray Analysis

Global examination of gene expression was performed with an Affymetrix HG-U133A array (Affymetrix, Santa Clara, CA, USA) using standard conditions (16 h, 45 °C) as described [1,2]. Arrays were washed and stained in a Fluidics Station 400 (Affymetrix) and scanned on a Gene Array Scanner 2500 (Agilent, Santa Clara, CA, USA). Raw fluorescence intensities from all hybridizations were normalized by applying variance stabilization with additional scaling. MAS5 and gcRMA expression values were calculated. Data and cluster analyses were performed using Affymetrix Microarray Suite 5.0 (MAS5) and GeneSpring GX software. Probes were made from total RNA following the guidelines given in the Affymetrix GeneChip Expression Analysis Technical Manual. An amount of 10 µg of fragmented, labelled cDNA was used for hybridization [1,2].

### 2.4. Clinical Data Analysis

Publicly available gene expression and survival data sets were obtained from the Cancer Genome Atlas (TCGA) filtering for patients with HNCs (TCGA HNSC). A total of *n* = 612 patients were included, and data were analyzed as described before [48]. For detailed patient characteristics, see also Supplementary Table S2. Data was assessed via the USCS Xena server [51] and patients were grouped according to indicated phenotypic or clinical characteristics. Final visualizations and statistical analyses were performed with Graphpad Prism. Furthermore, methylation analysis was performed using the MEXPRESS online tool [52]. Analysis of single-nucleotide variations was performed with the BioMuta v4.0 online tool [53].

### 2.5. Microscopy, Fluorescence Imaging, and Quantitation of Cells

Observation, quantitation, image analysis, and presentation were performed using Axiovert 200 M fluorescence microscope (Zeiss, Jena, Germany) as described [48,54,55]. To determine the average number of cells showing p-Akt staining, at least 200 fluorescent cells from three separate images were examined, visually inspected, and counted. Immunofluorescence staining of γH2AX was performed as reported in detail [48].

### 2.6. RNA Extraction, Reverse Transcription (RT)-PCR, and Quantitative Real-Time PCR Analysis

Total RNA was purified, and first-strand cDNA synthesis was carried out using a cDNA synthesis kit (Superscript II, Invitrogen life technologies). An amount of 1 µg of total RNA was converted to cDNA and 1 µL of the produced cDNA was amplified for 30 cycles (initial denaturation at 95 °C for 3 min, 30 s at 95 °C, 30 s at a variable temperature for annealing, and 1 min at 72 °C) followed by an extension of 5 min at 72 °C (for primer sequences please see Table S4). RT-PCR amplification products were analyzed on 2% agarose gels stained with ethidium bromide. GAPDH was used as a control.

Quantitative real-time PCR analysis was performed using the LightCycler^®^ 1.5 (Roche, Switzerland). PCR reaction mixtures consisted of 4 µL of LC FastStart DNA Master^plus^ SYBER^®^ Green Supermix (Roche, Basel, Switzerland), 50 ng of each target primer, and 2 µL cDNA template in a final reaction volume of 20 µL. Thermal cycling for OSF-2 was performed as follows: (95 °C, 600 s; 40 × (95 °C, 10 s; 62 °C, 5 s; 72 °C, 5 s); 72 °C to 95 °C melting curve analysis). Thermal cycling for RNA-PolII (housekeeping gene control) was performed as follows: (95 °C, 600 s; 40 × (95 °C, 10 s; 60 °C, 15 s; 72 °C, 15 s); 72 °C to 95 °C melting curve analysis). Cumulative fluorescence was measured at the end of the extension phase of each cycle. Specific amplicon formation with each primer pair was confirmed by melting curve analysis and by visualization of a single band on a 2% agarose gel. For primer sequences please see Table S4.

To define the relative expression of OSF-2 in CAFs, the results from the CAF sample were compared with the results from primary tumor cells. The relative expression ratio (R) of OSF-2 is calculated using the equation: Ratio = (E_target_)^ΔCp target(control-sample)^/(E_ref_)^ΔCpref(control-sample)^ based on its real-time PCR efficiencies (E = 1.5) and the crossing point (CP) difference of CAF sample versus PT and expressed relative to the non-regulated housekeeping gene (PolII), as described.

### 2.7. Study Population, Tissue Preparation, and Immunohistochemistry (IHC)

Investigations were conducted in accordance with the ethical standards according to the Declaration of Helsinki as well as according to local, national, and international guidelines as described [14]. Tissue samples were obtained from patients undergoing surgical resection at the department of otolaryngology of the Universities of Mainz. The study protocol has been approved by the local ethics committee (#83756604) after obtaining the patients’ informed consent to participate in the study and was processed anonymously. Cases were clinically and histologically diagnosed according to established criteria including grading and TNM classification [14]. All experiments were performed in accordance with relevant laws and the University Medical Center Mainz Guidelines and approved by the institutional ethics committee. In this study, tumor specimens, corresponding non-malignant tissue, and lymph node metastases were analyzed. Tissue samples were fixed and paraffin-embedded (FFPE) as described [56]. Tissues were processed for IHC as described [57,58]. Antigen retrieval was performed in a pressure cooker (sodium citrate, 10 mM, pH 6.0). For visualization of human OSF-2 protein (polyclonal α-OSF-2 Ab-diluted 1:150), the EnVision^®^ detection system (Dako GmbH) was used as described [57,58]. Sections were counterstained with hematoxylin. Negative control slides without primary Ab were included for each staining [56].

### 2.8. Plasmids and Sequence Analyses

To construct an OSF-2 expression plasmid, cDNA was isolated out of HNC cancer cell lines, and the full open reading frame of human OSF-2 cDNA was cloned into pcDNA3.1 mammalian expression vector (Invitrogen) with C-terminal GFP-tag (for primer sequences please see Table S4). The cloned variant of OSF-2 (referred to as full-length) corresponds to published isoform3 (Q15063-3) lacking exons 17 and 21. The plasmid was introduced into cells as described before [56,58]. Secretion-GFP was generated by the insertion of the OSF-2 secretion signal (aa1-21) into pc3-GFP. The OSF-2 secretion mutant ΔSec-GFP was amplified from full-length OSF-2-GFP and cloned into pc3-GFP using SacII/NheI-restriction sites. For sequencing the C-terminus of OSF-2, cDNA was amplified (for 5′-CACCTGACACCAGGAGTTTTC-3′; rev 5′-AAAGCTAGCCTGAGAACGACCTTCCCTTAATC-3′), cloned in a pGEM T easy Vector System (Promega, Madison, WI, USA), and several clones were sequenced (Genterprise, Mainz). RNA sequencing (RNASeq) was then performed as described in [59] and visualizations were achieved with the help of Graphpad Prism.

### 2.9. Protein Extraction, Immunoblot Analysis

Preparation of whole cell lysates was carried out as described using a low salt lysis buffer (50 mM Tris pH8.0, 150 mM NaCl, 5 mM EDTA, 0.5%NP-40, 1 mM DTT, 1 mM PMSF, complete EDTA-free from Roche Diagnostics, Mannheim, Germany) [58]. Preparation of cell lysates for phospho-staining was carried out according to the supplier’s recommendation. Subcellular fractionation was performed using the Qproteome Cell Compartment Kit (Qiagen, Valencia, CA, USA) according to the supplier’s recommendation. Fractions were analyzed by Western blotting. The purity of the fractions was determined using α-lamin B1 (nucleus), α-GAP-DH (cytosol), and α-Vimentin (membrane, cytoskeleton) Abs. equal loading of lysates was controlled by reprobing blots for housekeeping genes (actin, GAPDH). At least *n* = 2 biological replicates were performed and representative results are shown. Results of densitometric analyses of all Western blots can be found in the supplementary.

### 2.10. Cellular Assays

The scratch assay was performed using Ibidi culture inserts and the TScratch-software. Briefly, 7 × 10^3^ cells were seeded into each insert chamber, and after 24 h the insert was removed, the medium was replaced and pictures of the dish were taken every 30 min for 12 h.

To measure cell viability, cells were incubated with 100 ng/mL of-2 in 1% FCS-containing medium or 1% FCS-containing medium for 4 days. The medium was replaced every 24 h and cell viability was examined using the CellTiter-Glo Luminescent Cell Viability Assay (Roche, Mannheim) according to the manufacturer’s instruction. Luminescent signals were recorded using a Tecan Spark^®^ (Tecan, Männedorf, Switzerland) and normalized to untreated controls. For 3D assays, spheroids were grown in 96-well round bottom, ultra-low adhesion cell culture plates (Corning, New York, NY, USA; 1000 cells/well), and initial spheroid formation was allowed for 3 days. Spheroids were either treated (*n* = 4) with cisplatin and viability probed as described above, or observed for 10 days to determine clonogenicity.

For colony formation assay, 100 cells per ml were seeded in T-25 flasks (*n* = 24) and grown for 8–10 days. Colonies were fixed with 4% PFA, stained with Giemsa for 5 h at RT, washed with water, and counted using COLCOUNT™ (Oxford Optronix).

Matrigel invasion assay was performed using transiently transfected HeLa cells. A volume of 50–100 µL diluted matrigel (BD Bioscience, Billerica, MA, USA) was pipetted into the upper chamber of the 24-well transwell (Falcon BD, 8 µm pore size). Twenty-four hours after transfection, 1 × 10^5^ cells were seeded in 100 µL of cell suspension in a medium with 1% FCS into the upper chamber of the transwell. After gelling at 37 °C for at least 4–5 h, the lower chamber was filled with 10% FCS medium and incubated at 37 °C. Cells were observed for 4 days.

### 2.11. Xenograft Experiments

All animal work has been conducted according to relevant national and international guidelines. All animal experiments were approved by the Institutional Animal Care and Use Committee at the University of Mainz. Equal numbers (1 × 10^6^/animal) of stably OSF-2-GFP-expressing A431 cells and control cells were subcutaneously injected into both flanks of four-week-old female MRI nu/nu mice (Harlan Winkelmann, Hamburg, Germany) as described [1,60,61]. Six mice per group were used for three groups (Vector control/OSF-GFP wt/ΔSec-OSF-GFP). Tumor growth was monitored using calipers to calculate tumor volumes with the formula: length × π width^2^ × 0.52. Animals were euthanized at the end of the study.

### 2.12. Statistical Analysis

Statistical analyses were performed using Graphpad Prism (version 9.3.1) as described before [48]. Survival data were assessed via the USCS Xena server, visualized, and analyzed by Graphpad Prism (Log-rank/Mantel-Cox test; Hazard Ratio (Mantel-Haenszel)). For two groups, a paired or unpaired Student’s *t*-test, for more groups analysis of variance (ANOVA) was performed. Unless stated otherwise, *p* values represent data obtained from two independent experiments performed in triplicate. Statistical significance is represented in figures as follows: * *p* < 0.05, ** *p* < 0.01, *** *p* < 0.001, **** *p* < 0.0001, and n.s. indicates not significant. A *p*-value that was less than 0.05 was considered statistically significant.

## 3. Results

### 3.1. OSF-2 Is Overexpressed in HNC Primary Tumors and Lymph Node Metastases

HNC shows huge heterogeneity in their pathobiological and clinical behavior, which cannot yet be reliably predicted using current biomarkers. Hence, the identification of new biomarkers/drug targets to monitor disease progression, survival predictions, and therapies is of key interest [1,7,48,61,62].

To identify genes differentially expressed in HNC primary tumors (PT) versus lymph node metastases (LN M) and the corresponding non-malignant tissue (N), we report transcriptomics data from 15 patients undergoing surgical resection (for clinical and pathological characteristics and a list of differentially expressed genes see Supplementary Tables S1 and S3). In contrast to other studies, in which variations in individual patients require the analysis of a large number of unmatched samples to increase the relevance of the obtained data sets, our study focused on a patient cohort from which PT, N, and LN M could be obtained from the same patient. Notably, although the TCGA HNC data set comprises in total of 528 tumor tissues and 82 non-matched non-malignant tissues, the collection contains full experimental data from only two LN M, limiting the bioinformatic identification of pathways relevant for metastases.

**Figure 1:**
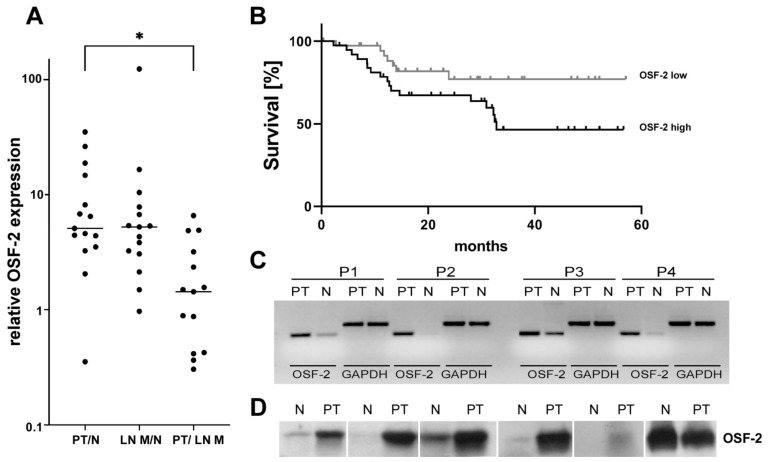
OSF-2 is significantly overexpressed in primary and metastatic HNCs. (**A**) RNA microarray analyses revealed at least 5-fold median overexpression of OSF-2 in the primary tumor (PT) versus normal tissue (N), as well as in lymph node metastases (LN M) versus N. PT versus LN M ratio indicated no further increase in OSF-2 expression in LN M (left). *n* = 15, * *p* < 0.05. (**B**) Survival analysis demonstrates that high OSF-2 expression levels correlate with reduced overall survival of HPV negative HNC patients; *n* = 75; *p* < 0.05. Hazard Ratio (Mantel-Haenszel) = 2.230. OSF-2 low < 12.3 (median), and OSF-2 high ≥ 12.3. (**C**) OSF-2 overexpression in PT versus N was confirmed by RT-PCR. OSF-2 up-regulation is shown in four representative cases (demographics see Table S1). GAPDH was used as a control. (**D**) Verification of OSF-2 microarray results on the protein level (*n* = 6). Higher expression of OSF-2 protein was found in PT versus N in five out of six representative cases. The uncropped western blot figures were presented in Figure S9.

In our data set, OSF-2 was identified among the top genes significantly up-regulated in PT versus N and LN M (Table S3; Figure 1A). Microarray results showed a metastases/normal (LN M/N) ratio that was even higher than the tumor/normal (PT/N) ratio (Figure 1A), suggesting the relevance of OSF-2 for metastases. We did not observe an additional increase in OSF-2 levels in PT/LN M, indicating that OSF-2 function seems to be important to maintain rather than to initiate metastatic growth. Although bioinformatic results are the first important step for the identification of potential biomarkers, it is necessary to carefully confirm obtained insights through independent experiments on the RNA and protein levels. Thus, RT-PCR, as well as Western blot analysis, was performed in tissues of our cohort used for microarray analyses, confirming OSF-2 up-regulation (Figure 1C,D).

When we further analyzed the expression and clinical relevance of OSF-2 by exploiting the *TCGA* HNC data set, comprising 612 cancer patients of various disease states and clinical backgrounds, we also found OSF-2 significantly upregulated in PT versus N (Supplementary Figure S1). As the HPV status influences clinical response and overall survival, we examined HPV-negative versus HPV-positive patients. Interestingly, high OSF-2 expression correlated with poor overall survival of HPV-negative HNC patients (*n* = 75; *p* = 0.05) (Figure 1B). Of note, these data support the transcriptomics data, which were also obtained from HPV-negative patients. In contrast, high OSF-2 expression did not correlate with poor survival in HPV-positive HNC patients (data not shown, *n* = 39; *p* < 0.05). However, caution is advised in this conclusion due to the small sample size available for the statistical analysis. We recommend additional investigations in larger patient cohorts in follow-up studies to evaluate the impact of HPV infection on OSF-2 expression and pathobiology.

Despite the wealth of clinical data, the TCGA HNC data set comprises transcriptomics data from only two LN M (patients are classified as N2a). Still, comparing OSF-2 levels in these samples supports our hypothesis that OSF-2 function seems to be important to maintain rather than drive metastatic growth (OSF-2 levels: TCGA-KU-A6H7-01 (PT) 9.56; TCGA-KU-A6H7-06 (matched LN M) 9.108; TCGA-UF-A71A-01 (PT) 14.23; TCGA-UF-A71A-06 (matched LN M) 13.63).

**Figure 2:**
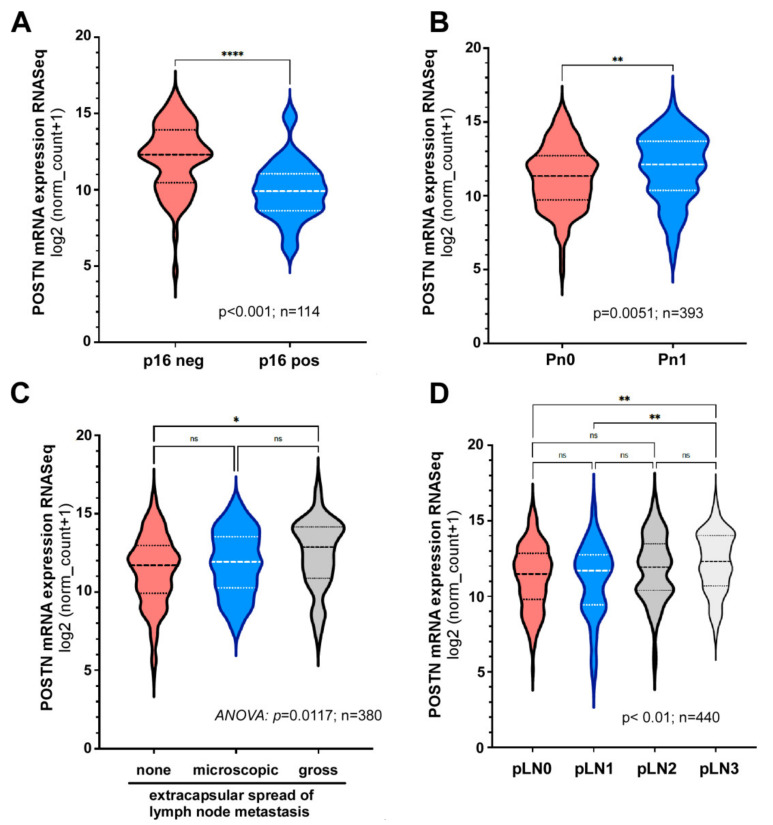
OSF-2 expression correlates with HPV status and markers of metastatic disease. Bioinformatic analysis of the *TCGA* HNC patient cohort (*n* = 612). High OSF-2 expression significantly correlates with (**A**) HPV status, (**B**) perineural infiltration, (**C**) extracapsular spread (ECE), and (**D**) pathological lymph node status (pLNx) for p16 negative or unknown. N2a/b/c and N3a/b are pooled, respectively; AJCC 8th Edition. *p*-values and sample size (*n*) as indicated. * *p* < 0.05, ** *p* < 0.01, **** *p* < 0.0001, and ns indicates not significant.

Interestingly, survival analysis indicated a correlation of OSF-2 expression with the HPV status of the tumor. OSF-2 was significantly increased in HPV negative compared to positive tumors (Figure 2A). Moreover, high OSF-2 expression correlated with markers of metastatic diseases, such as increased perineural infiltration, extracapsular spread, or lymph node metastases (pathological status; pLNx) (Figure 2B–D). Here, patients classified with pLN3 express significantly higher amounts of OSF-2 compared to pLN0 patients (Figure 2D). In contrast, OSF-2 expression did not correlate with HNC tumor localization, such as oral cavity, larynx, oro-, or hypopharynx (Supplementary Figure S2).

As DNA methylation belongs to crucial epigenetic mechanisms controlling transcriptional processes in many cancers, including HNC [20,63], we bioinformatically examined the OSF-2 gene methylation status. Using the MEXPRESS analysis tool [52], the data from the six CpG sites covered in the *TCGA* cohort (*n* = 612; PT = 528; LN M = 2; N = 82), demonstrated that PT showed significantly lower methylation levels compared to N correlating with increased OSF-2 expression in PT versus N (Supplementary Figure S3A). Furthermore, the OSF-2 methylation status correlated with the HPV status of the patients. Here, HPV-positive tumors exhibited increased CpG methylation (Supplementary Figure S3B). These results suggest that reduced gene methylation may at least be partially responsible for increased OSF-2 levels in HPV negative HNC tumors and non-malignant tissue.

In addition to OSF-2, also other matricellular proteins, such as osteopontin (SPP1), were suggested to impact HNC tumorigenesis and metastases [28,29]. However, in contrast to OSF-2, we did not observe a significant correlation between osteopontin expression with overall patient survival (Supplementary Figure S4). Consequently, we here focused on OSF-2 in our experimental pipeline.

### 3.2. OSF-2 Expression Does Not Directly Contribute to Therapy Resistance

**Figure 3:**
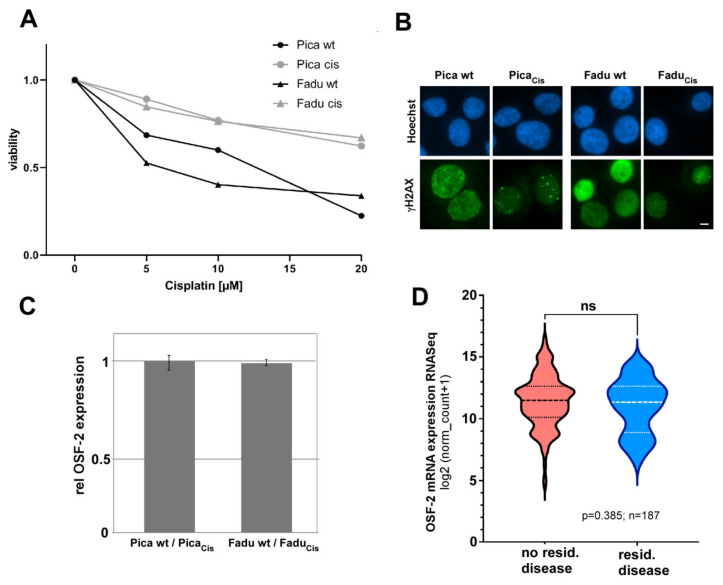
OSF-2 expression does not directly contribute to cisplatin therapy resistance. (**A**) Selected cisplatin-resistant Pica_Cis_ and FaDu_Cis_ cells (grey) are highly resistant. Cells were treated with indicated cisplatin concentrations for 48 h and viability normalized to untreated controls. (**B**) Resistant Pica_Cis_ and FaDu_Cis_ cells show a lower number of cisplatin-induced DNA damage foci (γH2AX) compared to wt Pica/FaDu cells. Cells were treated with 20 µM cisplatin and analyzed by fluorescence microscopy after 24 h. Scale bar, 5 µm. (**C**) OSF-2 is not overexpressed in resistant Pica_Cis_/FaDu_Cis_ versus sensitive wt cells. OSF-2 expression was quantified by RNASeq transcriptomics; relative mRNA expression is shown. (**D**) OSF-2 expression does not correlate with locoregional remission status after primary therapy (*disease after curative treatment)* in the *TCGA* HNC patient cohort. *p*-value and sample size (*n*) as indicated.

First-line chemotherapy in HNC is mainly cisplatin-based [2,48,64,65,66]. It has been suggested that OSF-2 also contributes to the development of cisplatin resistance in cancer, such as lung cancer [67], although the underlying mechanisms are not understood. To experimentally investigate the potential impact of OSF-2 on chemoresistance in HNC, we established two cisplatin-resistant HNC cell lines allowing the comparison of cisplatin-sensitive versus -resistant cells (Pica_Cis_ and FaDu_Cis_; Figure 3). Although the cells were highly resistant to cisplatin, as shown in 2D (Figure 3A) as well as 3D spheroid cultures (Supplementary Figure S5), NGS RNA sequencing transcriptomics did not reveal an up-regulation of OSF-2 in resistant versus the sensitive parental HNC cell lines (Figure 3C). Moreover, bioinformatic analysis of the HNC *TCGA* patient cohort failed to identify a correlation of OSF-2 expression with residual disease after first-line, predominantly platinum-based, chemo(radio)therapy (Figure 3D). These data strongly suggest that OSF-2 expression appears not to directly contribute to therapy resistance in HNC. However, we cannot exclude indirect effects, e.g., by up-regulation of anti-apoptotic protein, such as Survivin [57,68,69].

### 3.3. OSF-2 Is Overexpressed in the Tumor Microenvironment

**Figure 4:**
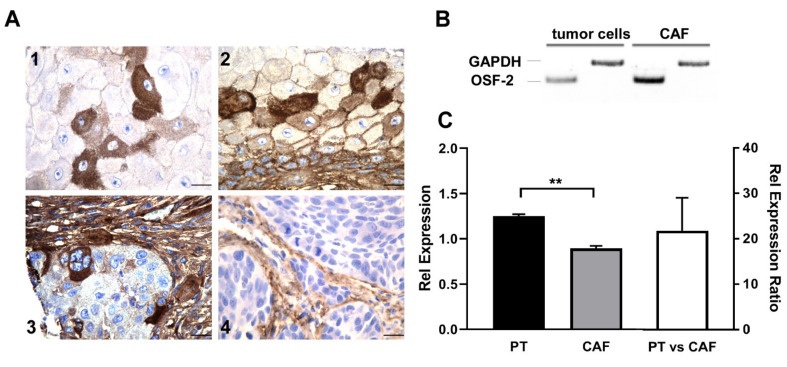
OSF-2 is expressed in the tumor microenvironment. (**A**) Immunohistochemical analysis of OSF-2 expression in primary HNC tissue. Intense staining was observed in cancer cells (**1**,**2**), but also in the juxtatumoral stroma (**3**,**4**). Scale bars, 20 µm. (**B**) RT-PCR of primary tumor cells and CAFs revealed higher expression of OSF-2 in CAFs. GAPDH was used as a control. (**C**) Up-regulation of *OSF-2* in CAFs compared to primary tumor cells was confirmed by quantitative real-time-PCR. Up-regulation is shown by lower C_T_ values and thus, a lower relative expression for CAFs. OSF-2 expression in primary tumor cells (PT, black) and CAFs (grey) is shown compared to RNA-Pol II expression (OSF-2/RNA-Pol II; left axis), and as relative expression ratio R (right axis). R = 21.18, *n* = 2, ** *p* < 0.005. The uncropped western blot figures were presented in Figure S10.

To further examine OSF-2 expression on the protein level, we performed immunohistochemical (IHC) staining of HNC tissue sections (Figure 4). Interestingly, IHC staining detected OSF-2 not only in the cytoplasm and the granulae in tumor cells (Figure 4(A1)) but also in the surrounding stroma (Figure 4(A3,A4)). Moreover, high OSF-2 levels were also detectable in the intercellular space, indicating protein secretion (Figure 4(A2)). Indeed, when we examined cancer-associated fibroblasts (CAFs) by quantitative RT-PCR, and real-time PCR, we found a more than 20-fold overexpression of OSF-2 (relative expression ratio R = 21.18) in CAFs compared to tumor cells, supporting our hypothesis that OSF-2 expression/secretion appears to generate a tumor-friendly microenvironment (Figure 4B,C).

### 3.4. HNC Patients Express Various OSF-2 Isoforms

**Figure 5:**
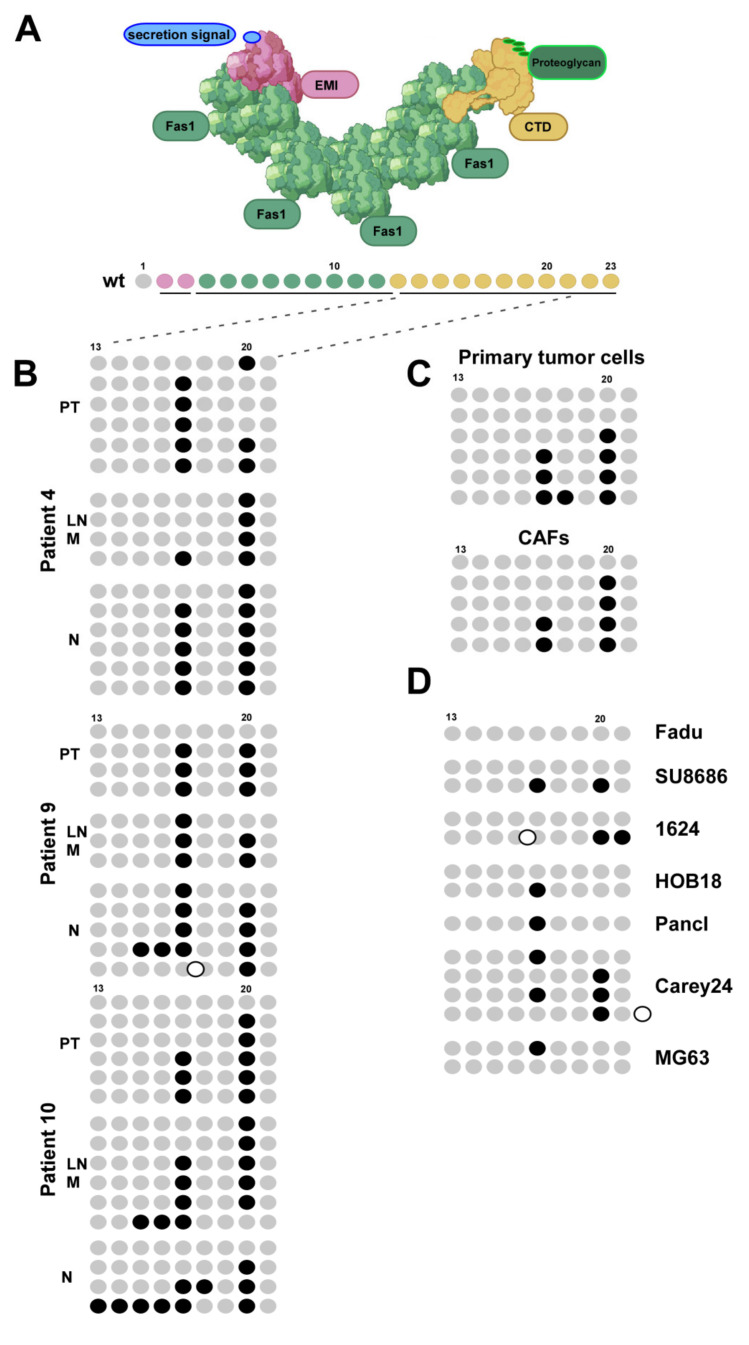
HNC patients express various OSF-2 isoforms differing in their C-terminal domain. (**A**) Proposed protein domain organization of OSF-2 (modified from [40]). The canonical sequence (wt) consists of 23 exons building the N-terminal secretion signal (blue, identified in this study), the EMI domain (red, exon 2–3), four Fas1 domains (green, exons 3–13), and the variable C-terminal domain (CTD, yellow, exons 13–23). (**B**) Expression of OSF-2 isoforms was examined by sequence analyses of the OSF-2 C-terminus (exon 13–21), and compared to the ‘canonical’ sequence (wt). In total, 12 isoforms were identified. Grey dots represent verified exons, black dots lack exons (deletions), and white dots have additional exons (insertions). OSF-2 isoform distribution was analyzed in multiple clones of 3 patients (PT, LN M, N) from our study cohort (**B**), isolated primary tumor cells and corresponding CAFs (**C**), as well as in different HNC tumor cell lines (**D**).

Although a ‘canonical’ coding sequence for OSF-2 (Q15063-1) has been deposited [38], there is an ongoing debate if alternative isoforms, potentially executing additional functions, are expressed in (HNC) tumor patients. To address this question, we cloned OSF-2 coding sequences from primary tissues. Interestingly, sequence analysis revealed that the OSF-2 isoforms we found significantly differ from other variants previously reported for other tumor types [37,38,70] (Figure 5). To further examine isoform expression signatures, we analyzed the sequence of the OSF-2 C-terminus (exon 13-21, aa 569-789) in different cell lines, primary tumor tissue, corresponding lymph node metastases, normal tissue, and tumor-associated fibroblasts (Figure 5).

Surprisingly, we did not detect the ‘canonical’ sequence of OSF-2 (Q15063-1) in the analyzed samples, but instead, 12 OSF-2 isoforms exhibited deletion of exon 17 and/or exon 20 and thus, differing in their C-terminus. The distribution of isoforms was not specific for malignant versus normal cells. Interestingly, an inspection of the proposed OSF-2 protein domain structure suggested that these deletions within the C-terminal domain (CTD) may affect postulated OSF-2 functions, such as regulation of extracellular matrix and multimerization [71,72]. However, these proposed functions are not yet understood mechanistically. The wide appearance of these isoforms in patients’ tissue however indicates the relevance of the CTD domain, which needs to be clarified in future studies. Our findings indicate also for the field that when studying molecular OSF-2 functions, caution may be advised to exclusively focus on the ‘canonical’ OSF-2 sequence (Q15063-1) but underline the need to investigate OSF-2 forms really expressed in the respective disease tissues. Notably, one may suggest probing the biological functions of OSF-2 variants by means of recombinant proteins. The commercially available recombinant OSF-2 protein (rOSF-2) used here is produced in *E. coli* and was also used in other studies [73,74]. rOSF-2 is comparable to natural occurring OSF-2 variants we found in HNC tumors also lacking exons 17 and 20 (Figure 5). Potentially, one could consider using rOSF-2, encoding the isoforms we identified in our study, to (fine)map so far unknown biological functions of OSF-2, e.g., of the CTD domain. However, as OSF-2 is glycosylated (FAS1 domain) in cells, we advise caution that rOSF-2 produced in *E. coli* will be the most adequate tool to identify all potential functions.

As shown for many HNC-relevant proteins, (in)activating mutations are often found in tumor cells. Thus, we further investigated the mutation frequency and type in OSF-2 by exploiting the BioMutav3.0 database and data provided by the TCGA HNC collection. Here, the analysis of 507 HNCs from the TCGA cohort showed a low number of mutations in patients, indicating again that various OSF-2 isoforms may be active in cancer patients (Supplementary Figure S6).

### 3.5. OSF-2 Isoforms of HNC Patients Contain an Evolutionary Conserved, Functional Secretion Signal

**Figure 6:**
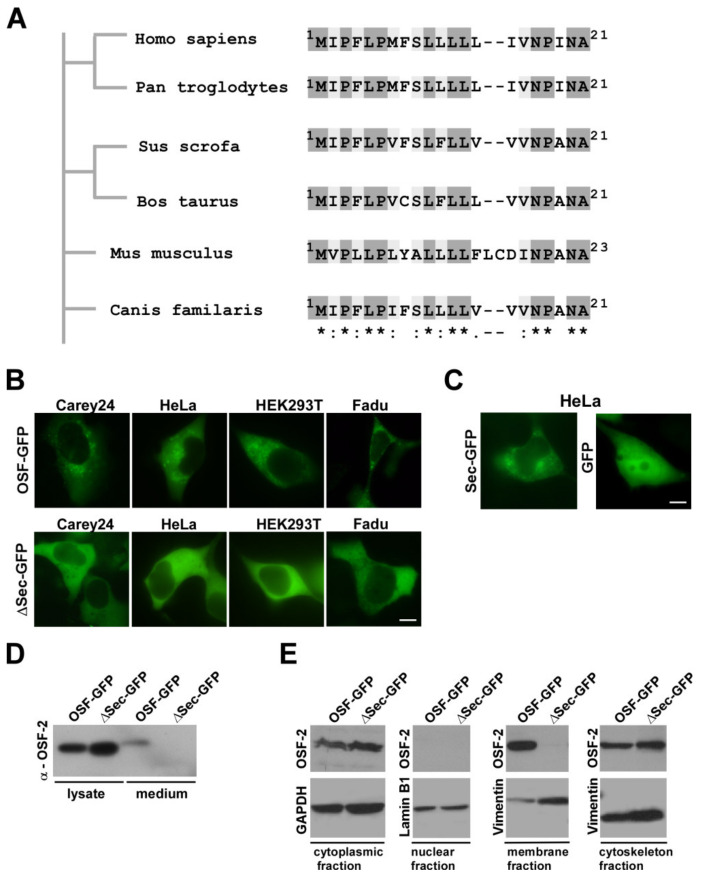
OSF-2 contains an evolutionarily conserved secretion signal. (**A**) Alignment of predicted secretion sequences in various OSF-2 homologs. Phylogram constructed on the basis of amino acid sequence similarities depicting the evolutionary relationships among OSF-2 proteins of different species. The sequence of the predicted human secretion signal (aa1-21) is conserved in all compared homologs. * Identical residues (dark grey), conserved substitutions/similar characteristic (light grey), semi-conserved substitution/similar shape (white). Organisms and amino acid positions are indicated. (**B**) OSF-2-GFP transfection in different tumor cell lines revealed a cytoplasmic granular localization. No secretion granulae were observed upon expression of the secretion mutant, ΔSec-GFP. (**C**) Expression of the signal alone fused to GFP (aa1-21; Sec-GFP) was sufficient for the formation of secretion vesicles. GFP expression served as the negative control. Scale bars, 5 µm. (**D**) Western blot confirming OSF-2 secretion. (**E**) Immunoblot analysis of cell fractions from OSF-2-GFP and ΔSec-GFP HEK293T transfectants. Only OSF-GFP was detectable in the membrane fraction of secretion vesicles and the supernatant. In contrast, the OSF-ΔSec-GFP secretion mutant failed to be incorporated into vesicles or to be secreted. Probing with anti-GAPDH (cytoplasm), anti-Vimentin (membrane, cytoskeleton), and anti-Lamin B1 (nuclear) Abs served as controls for lysate preparation. Representative results for *n* = 2 are shown. The uncropped western blot figures were presented in Figures S11 and S12.

As IHC analysis revealed high amounts of extra/inter-cellular OSF-2, suggesting active protein secretion, we searched for a potential secretion signal in the OSF-2 protein sequence. Global protein alignment of homologous OSF-2 proteins from *Homo sapiens*, *Pan troglodytes* (chimpanzee), *Sus scrofa* (pig), *Bos taurus* (cattle), *Mus musculus* (mouse), and *Canis familaris* (dog) revealed overall protein similarity ranging between 90 and 96.5% and proposed the first ~21 aa as a putative secretion signal (Figure 6A). However, bioinformatic predictions need to be confirmed experimentally. Hence, we first engineered a GFP-tagged full-length OSF-2 protein (OSF-GFP), the isolated proposed secretion signal fused to GFP (aa1-21; Sec-GFP), and a full-length mutant lacking the signal (OSF-ΔSec-GFP). The functionality of the suggested signal in living tumor cells was demonstrated by independent evidence: First, expression of OSF-2-GFP in different tumor cell lines showed a cytoplasmic granular localization, typical for secreted proteins (Figure 6B). No OSF-GFP was present in the nucleus. Second, no secretion vesicles were evident upon expression of the OSF-ΔSec-GFP secretion mutant. Third, the expression of the signal alone fused to GFP (aa1-21; Sec-GFP) was sufficient for the formation of secretion vesicles (Figure 6C). Moreover, in subcellular fractionation experiments, only OSF-GFP was detectable in the membrane fraction of secretion vesicles and the supernatant (Figure 6D,E). In contrast, the OSF-ΔSec-GFP secretion mutant failed to be incorporated into vesicles or to be secreted. Thus, we here identified the OSF-2 aa 1-21 (^1^MIPFLPMFSLLLLLIVNPINA^21^) as its *bona fide* secretion signal.

### 3.6. OSF-2 Does Not Affect Tumor Cell Proliferation but Is Crucial for Cell Migration and Cellular Survival under Stress Conditions

**Figure 7:**
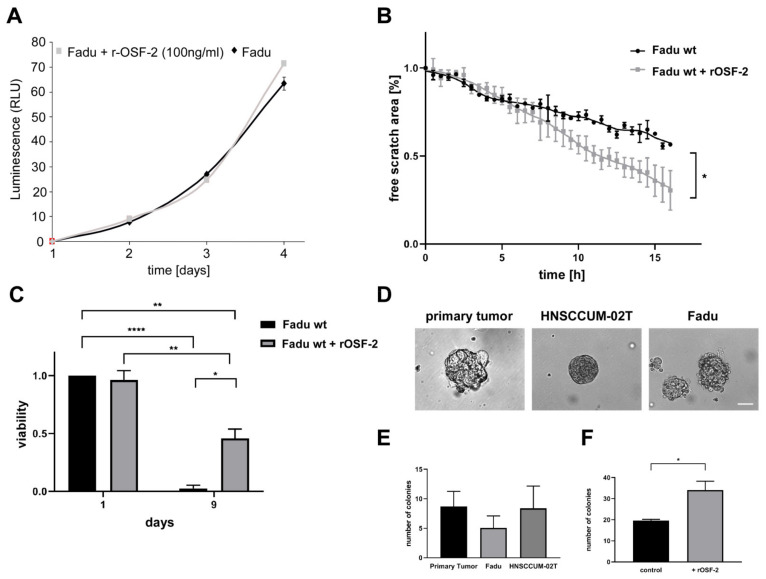
OSF-2 promotes cell migration and cellular survival under stress conditions. Administration of recombinant OSF-2 (100 ng/mL) did not affect HNC cell (FaDu) proliferation (**A**), but resulted in increased cell migration (**B**), and improved survival under serum deprivation (**C**). (**D**,**E**) Spheroid and colony formation assays revealed clonogenicity of three OSF-2 overexpressing HNC cell lines (controls without treatment). Scale bar, 100 µm. (**F**) Presence of recombinant OSF-2 increases the number of formed colonies using Fadu cells. *p*-values of unpaired *t*-testing as indicated. * *p* < 0.05, ** *p* < 0.01, **** *p* < 0.0001.

Enhancing tumor metastasis can be influenced by increasing (local) tumor cell proliferation or/and by increasing the cell’s capability to migrate and survive in distant ‘hostile’ microenvironments. Thus, we performed in vitro and in vivo proliferation and scratch assays. Intriguingly, the administration of recombinant OSF-2 (100 ng/mL) did not affect cell proliferation (Figure 7A). Likewise, ectopic OSF-2 overexpression in transfectants did not affect local tumor growth in xenograft models (Supplementary Figure S7). In addition, ectopic expression of secretion active versus secretion-deficient OSF-2 did not significantly enhance invasion in matrigel assays (Supplementary Figure S8). In contrast, the administration of recombinant OSF-2 significantly increased cell migration (Figure 7B) and cellular survival under serum starvation ‘stress’ conditions (Figure 7C). To further mimic ‘hostile’ metastases microenvironments, we employed clonogenic 3D-spheroid formation assays (Figure 7D–F). Here, OSF-2 expressing, patient-derived primary HNC tumor cells (characterized in Figure 4B,C and Figure 5C), as well as two HNC cell lines, were cultivated as 3D-spheroids (Figure 7D,E). Treatment with recombinant OSF-2 significantly increased the number of formed spheroid colonies (Figure 7F).

Collectively, in contrast to other factors such as matrix proteases, promoting metastasis by enhancing invasion, these results strongly suggest that OSF-2 (over)expression promotes metastasis in head and neck cancer by acting on cancer cells and/or CAFs ectopically rather than by stimulating invasion or proliferation pathways intrinsically.

### 3.7. OSF-2 Promotes Cells by β1-Integrin-Induced Activation of the PI3-Kinase/Akt/PKB Pathway

To define the mechanisms of how OSF-2 promotes cellular migration and survival, we examined cancer-relevant signaling pathways. It has been suggested that OSF-2 stimulates Wnt signaling in breast cancer [75]. However, when we examined Wnt expression in metastases we did not detect a significant up-regulation. Whereas no differential expression of Wnt1 or Wnt3A could be detected we even found a down-regulation of Wnt-5A or -5B M versus N as well as LN M versus PT (see Supplementary File microarray analysis all). Moreover, Wnt expression did not correlate with patterns of metastatic disease in the HNC TCGA cohort.

**Figure 8:**
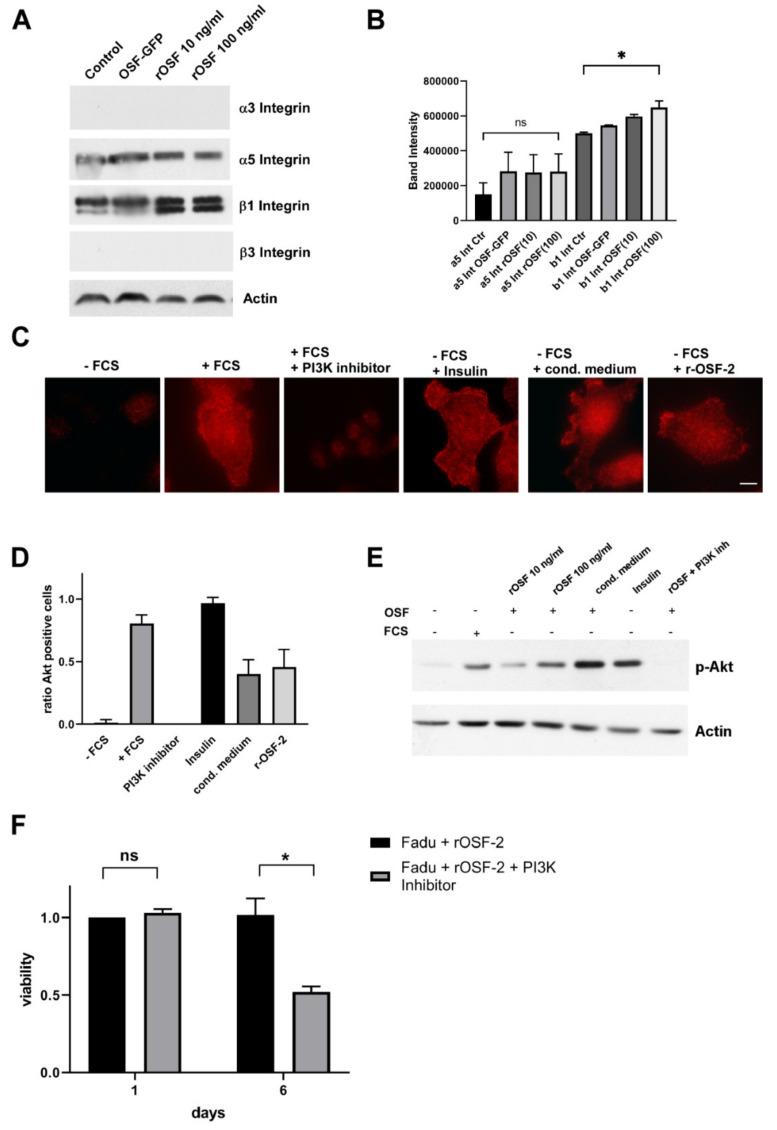
OSF-2 stimulates the Akt/PKB pathway via integrin-dependent PI3-Kinase activation. (**A**,**B**) β1-integrin, but not α5 integrin is induced by the addition of rOSF-2 (10, 100 ng/mL). Integrin receptor expression was analyzed by Western blot in HNSCCUM-02T cells. Actin served as the loading control. Representative results (*n* = 2) are shown. Densitometric analysis of detected bands is shown in (**B**). (**C**) Specific phosphorylation of Akt1/PKBα on Ser473 was detected in HNSCCUM-02T cells. Insulin and serum-containing medium (+FCS) served as positive, serum deprivation (−FCS), and PI3K-inhibitor (LY294002; +FCS) treatment as negative controls for staining. Scale bar, 5 µm. (**D**) Quantification of p-Akt positive cells. At least 200 cells from three separate images were examined, visually inspected, and counted as p-Akt positive or negative. Ratio of p-Akt positive cells (= #pAkt positive/all counted cells) is shown for indicated treatments. All treatments, except PI3K-inhibitor, and +FCS, were performed under serum starvation conditions. (**E**) Akt1/PKBα phosphorylation was confirmed by Western blot analysis using HNSCCUM-02T cells. Akt1/PKBα phosphorylation was induced by recombinant OSF-2 (rOSF-2) and conditioned medium under serum-free conditions. OSF-2-induced phosphorylation was prevented by PI3K inhibition. Actin served as the loading control. Representative results (*n* = 2) are shown. (**F**) HNC cancer cell (Fadu) survival was reduced after treatment with r-OSF-2 and a PI3K-inhibitor (LY294002). ns: *p* > 0.05; * *p* ≤ 0.05. The uncropped western blot figures were presented in Figure S13.

Next, we examined ECM/focal adhesion receptors in HNC. Notably, we found that β1-integrin, but not α3/α5- or β3-integrins was induced by the addition of rOSF-2 (Figure 8A,B), suggesting its potential role as an ‘OSF-2 receptor’. Indeed, treatment with a conditioned medium from engineered OSF-2 overexpressing cells as well as recombinant OSF-2 (r-OSF-2) did not only increase β1-integrin expression (Figure 8A) but also activated the Akt/PKB signaling pathway, as shown by immunofluorescence staining of phosphorylated Akt1/PKBα (Ser473) (Figure 8 C,D). Phosphorylation, indicative of Akt activation, was independently confirmed by Western blot analysis (Figure 8E). Furthermore, the positive effect of r-OSF-2 treatment on cellular survival under stress conditions could be counteracted by treatment with the PI3K-inhibitor LY294002 (Figure 8F). These results demonstrate that the effects of OSF-2 on cell migration and survival depend (at least) on integrin-mediated Akt/PKB signaling. Of note, serum OSF-2 levels in the range of 100 ng/mL have been reported [76,77,78]. Hence, although the exact local OSF-2 concentrations in the tumor microenvironment in vivo are not known, 10/100 ng/mL rOSF-2 represent realistic physiological doses, underlining the relevance of our findings.

## 4. Discussion

OSF-2 is investigated as a relevant multifunctional protein exhibiting executing various biological functions in health and disease, including cancer/metastasis, development, repair, and/or (bio)material tolerance [28,79]. However, since both tumor-promoting, as well as tumor-suppressive activities have been reported, their relevance and functional role in (head and neck) cancer are still controversially discussed. Thus, this study aimed at a clinical and functional characterization of OSF-2 in a cohort of metastatic head and neck tumors to assess its potential as a prognostic/diagnostic biomarker and/or as a target for novel therapeutic interventions.

First, our bioinformatic analyses revealed OSF-2 not only as significantly upregulated in primary tumors, as well as lymph node metastases, but also suggests OSF-2 as a potential prognostic biomarker for HPV negative head and neck tumors. Although it is widely accepted that HPV negativity correlates with poor survival, there is still a lack of robust biomarkers and molecular understanding for this subgroup. Interestingly, Martinez et al. proposed potential biomarkers correlating with patients’ HPV status [80]. Inspection of these data revealed that OSF-2 was among the differentially expressed genes, independently underlining the relevance of our finding. In contrast to OSF-2, another potential promotor of metastases we found upregulated in HPV negative tumors, osteopontin (*SPP1*), seems to play only a minor role in the clinical outcome of these patients. Our finding that high OSF-2 correlates with clinical parameters of high tumor aggressiveness in HNC, such as lymph node status and perineural infiltration, seems to be relevant also for other entities [44,81,82].

Additionally, we analyzed *OSF-2* gene methylation in the TCGA cohort of HNC patients to mechanistically understand OSF-2 up-regulation in primary tumors and metastases. Here, tumor tissues showed significantly lower methylation levels compared to normal solid tissues which are consistent with other methylation studies of HNC [1,20]. Thus, our data indicate that reduced gene methylation might at least be partially responsible for increased OSF-2 levels observed in tumors. However, due to the very small sample size of metastatic (lymph node) tissue in this cohort (*n* = 2), we could not draw conclusions about the up-regulation of OSF-2 in distant metastases. Results might also be limited due to the use of the *Infinium Human Methylation450 BeadChip* by *TCGA* Research Network which only covers a fraction of CpG sites within the region of interest. Further prospective studies applying additional experimental methods are needed, ideally exploiting matched normal, tumor, as well as metastatic tissues, also from distant sites, which though rare, are in HNC.

It has been suggested that OSF-2 (in)directly contributes to cisplatin resistance in lung cancer cells via activation of Stat3, Akt, and up-regulation of the anti-apoptotic protein survivin [67]. Furthermore, the contribution of OSF-2 to chemoresistance was indicated for ovarian carcinoma [83,84,85], and osteosarcoma [86]. Interestingly, whereas it has been indicated that OSF-2 affects to radioresistance of HNC cells [79], to our knowledge there are no studies about the role of OSF-2 in cisplatin-resistance of HNC. In contrast, the matricellular protein osteopontin was already suggested to be involved in cisplatin resistance and poor clinical outcomes of oral cell carcinoma [30]. However, our study revealed that OSF-2 expression was neither increased in two engineered cisplatin-resistant HNC cell lines nor correlated with residual disease after first-line therapy in the *TCGA* cohort. Of note, such controlled approaches have not been performed for other tumor types [67,79,83,84,85,86], precluding to conclude that OSF-2 directly causes therapy resistances. Our results suggest that OSF-2 does not directly induce therapy resistance (in HNC).

The tumor microenvironment does not only consist of neoplastic tumor cells but also of non-neoplastic stromal cells including fibroblasts, cancer-associated fibroblasts (CAFs), endothelial cells, pericytes, and inflammatory cells. Here, CAFs are building up the majority of the cellular tumor stroma and play an important role in tumor initiation, progression, and metastasis [87]. Especially for HNC, it has been reported that CAFs are key players in invasion, cancer relapse, treatment resistance, and poor patient outcomes [88]. It is still under debate if secreted OSF-2 is predominantly expressed directly by tumor cells or tumor stromal cells [89]. In vivo, two reports demonstrated that *OSF-2* overexpression in tumor cell lines increases metastases and angiogenesis in nude mice and reduces stress-induced apoptosis [44,90], while another report provided evidence that OSF-2 suppresses lung metastasis of mouse melanoma cell line B16-F10 [91]. Although OSF-2 overexpression does not seem to be systematic in human tumors, studies agree on the low level of OSF-2 expression in most tumor cell lines [91,92,93,94,95]. Lower levels of *OSF-2* expression in tumor cell lines compared to tumor tissues are in agreement with studies showing the production of OSF-2 by stromal cells—and not cancer cells—in tumors [81,82,96,97]. However, the nature of OSF-2-producing cells in tumors is another matter of controversy as separate in situ hybridization experiments suggested that *OSF-2* mRNA is detected in the cytoplasm of cancer cells [45,90]. Thus, in this study, we did not only analyze patient tumor samples but also isolated primary tumor cells, as well as CAFs to characterize OSF-2 expression and localization in different cell types. By immunohistochemical analyses we identified OSF-2 expression in both, tumor and stromal cells, the latter exhibiting more than 20-fold higher OSF-2 levels compared to tumor cells. It has to be mentioned that OSF-2 expression shows high variations between different cells. However, in contrast to other studies [45,81,82,96,97,98], it can be postulated that both tumor cells and tumor stromal cells contribute to the overall OSF-2 expression of HNC tumors. It will be relevant to further examine OSF-2 expression in CAFs and cancer cells also in samples of lymph node metastases via IHC in larger cohorts in a comprehensive follow-up study, including its impact on HNC disease.

Although the expression of OSF-2 isoforms produced by alternative splicing has been reported [38], little is known about their specific biological functions and the existence of cell/tissue-specific expression patterns. Here, we identified 12 isoforms of OSF-2 protein differing in their C-terminus. Importantly, we could not determine a cancer cell-specific expression signature of OSF-2 in HNC patients, primary cells, or cancer cell lines, but the deletion of exons 17 and 20 occurred with high incidence. The in-frame insertions and deletions of the detected isoforms suggest their generation via alternative splicing mechanisms as described before [37,38]. The fact that we did not detect expression of the postulated ‘canonical’ or wild-type sequence of OSF-2 (Q15063-1) consisting of 23 exons in all analyzed samples strongly suggests that this isoform does not represent the biologically active, predominant variant. Our findings indicate for the field that when studying molecular OSF-2 functions, caution may be advised to exclusively focus on the ‘canonical’ OSF-2 sequence (Q15063-1) but investigate OSF-2 expression signatures in the respective disease phenotype. Of note, recombinant OSF-2 protein used in this study also lacks exons 17 and 20 and thus, is comparable to natural occurring OSF-2 variants in HNC tumors.

Determining its subcellular localization and thus also its biological functions, the functional protein domains of OSF-2 were analyzed. Bioinformatic analyses suggest that OSF-2 contains an N-terminal secretory signal peptide, followed by a cysteine-rich domain, four internal homologous Fas1 repeats, and a C-terminal hydrophilic domain. Interestingly, we could show an abundance of OSF-2 in the intercellular space suggesting secretion of OSF-2 which we proved in this study for the first time also biochemically by secretion-deficient mutants. Additionally, subcellular localization studies revealed that deletion of the N-terminal secretion signal results in cytoplasmic and cytoskeletal localization, but not in membranous localization of OSF-2. Thus, we could prove that the secretion signal is exclusive and indispensable for the secretion of OSF-2 into the intercellular space. It has to be mentioned that the recombinant OSF-2 protein used in our study lacks the identified N-terminal secretion signal. However, for the conducted experiments the lacking secretion signal is negligible because ectopically rOSF-2 treatment mimics the naturally occurring secretion process of endogenous OSF-2 by tumor cells and/or CAFs. Interestingly, OSF-2 was identified as a protein-enriched also in exosomes secreted by metastatic breast cancer cells [99,100]. These results suggest that OSF-2 might execute its pro-migratory functions not only directly via up-regulation in (metastatic) cancer cells, and direct secretion into the intercellular space, but also by exosomal transfer to recipient cells. However, we did not detect OSF-2 protein in isolated exosomes from head and neck cancer cell lines (data not shown). Nevertheless, future analyses of OSF-2 abundance in exosomes derived from HNC cancer patients and other malignancies may help to evaluate its clinical relevance as a novel ‘liquid biopsy’ marker for metastatic disease [24,50].

**Figure 9:**
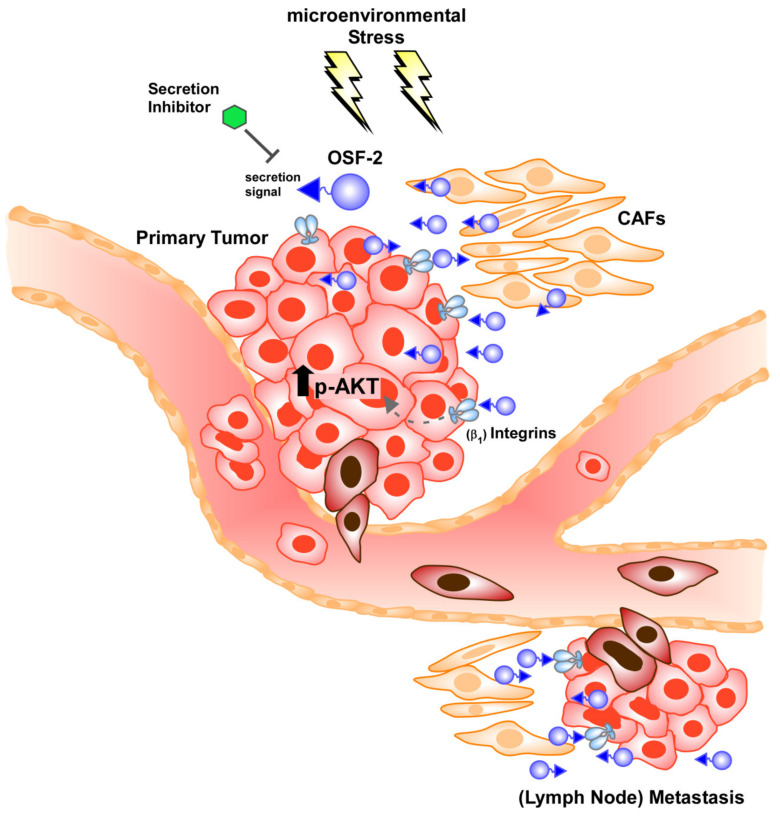
Proposed OSF-2 function in promoting (lymph node) metastases by regulating the tumor microenvironment and cellular survival. OSF-2 is overexpressed in tumor cells, as well as in cancer-associated fibroblasts (CAFs) at the primary tumor and lymph node metastatic sites in HNC. Though, it is likely that these mechanisms are also relevant for distant site metastases in other cancer types. Due to the conserved N-terminal secretion signal, OSF-2 seems to execute its tumor-promoting functions not mainly intracellularly, but extracellularly in the tumor microenvironment. Here, secretion inhibitors could be therapeutically relevant. OSF-2 modulates the tumor microenvironment by integrin-dependent activation of Akt/PKB pathways and downstream signaling, particularly critical under stress conditions.

Tumor metastasis can be influenced by a variety of factors, such as increasing (local) tumor cell proliferation, enhancing cancer cells’ invasiveness, increasing the cells’ capability to migrate, or, as only recently accepted, the ability to survive in distant ‘hostile’ microenvironments. However, in contrast to cancer-associated proteases, OSF-2 does not perform enzymatic proteolytic degradation per se. Though, there is indeed an intense discussion that OSF-2 promotes invasion indirectly by cross-talk with various signaling pathways, such as WNT, fibronectin, and TGFβ, thereby promoting angiogenesis and micrometastatic outgrowth [75,101]. The relevance of ectopic OSF-2 for the tumor microenvironment was supported by our studies on cell migration, clonogenic 3D-spheroid formation assays, and survival under stress conditions. Here, the data strongly suggest that OSF-2 overexpression acts on cancer cells and/or CAFs extrinsically via stimulation of integrin receptors, rather than by activating proliferation and pro-survival pathways, such as Akt/PKB signaling intrinsically (Figure 9). We propose that OSF-2-mediated regulation of the tumor microenvironment occurs especially under stress conditions (such as hypoxia, nutrient deprivation, pH changes, or reduced vascularization) by integrin-mediated activation of Akt/PKB pathways and downstream signaling.

It has been suggested that OSF-2 stimulates Wnt signaling in breast cancer (stem) cells [75]. However, when we examined Wnt expression in metastases we neither detected a significant up-regulation, nor a correlation with patterns of metastatic disease in the HNC TCGA cohort. Indeed, whereas breast cancer is a highly metastatic disease particularly also to distant organs, metastases of HNC are mainly found in local lymph nodes and metastases to distant organs are rare. Moreover, in contrast to breast cancer, we clearly detected OSF-2 overexpression also in primary tumor cells. Thus, OSF-2 induction of Wnt might be different in breast cancer versus HNC but clearly may deserve future investigations.

Due to its proposed multiple functions in proliferation, survival, stress response, invasion, and chemoresistance, OSF-2 also represents a promising therapeutic target for novel anti-cancer therapies. Small molecules directly targeting OSF-2 or integrin receptors and/or secretion inhibitors might be used to inhibit OSF-2-mediated metastasis and/or chemoresistance. However, such compounds still await their identification, and the field may be stimulated by the findings reported here. Moreover, to our knowledge, no small molecules altering OSF-2 expression have been reported. For breast cancer models, different approaches have been studied to inhibit OSF-2 activity. Lee et al. suggested that DNA aptamers directed against human OSF-2, and could efficiently block tumor growth and cell dissemination in a xenograft mouse model [102]. Recently, a peptide antagonist was engineered to overcome OSF-2-mediated tumor-promoting effects including chemoresistance in breast cancer [103]. Additionally, anti-OSF-2 antibodies have been used to inhibit tumor growth in vivo [104]. However, the mechanistic effects of these approaches are not always fully understood, and potential side effects on regenerating non-malignant tissues/bones need to be monitored carefully.

## 5. Conclusions

Metastasis is the main cause of cancer-related deaths and is responsible for about 90% of cancer mortality, including HNC. Our study demonstrates the (clinical) relevance of the osteoblast-specific factor 2 (OSF-2), predominantly for HPV-negative HNC patients. We show that OSF-2 executes its tumor-promoting functions mainly via the tumor microenvironment, rather than by stimulating proliferation and pro-survival pathways intrinsically. By identifying a secretion signal, we suggest not only exploiting OSF-2 as a potential prognostic biomarker for head and neck cancers but also targeting its tumor microenvironment reservoir by secretion inhibitors.

## Data Availability

The datasets supporting the findings of this study are indicated in the article and are available from the corresponding author on request.

## Supplementary Materials

The following are available online at https://www.mdpi.com/article/10.3390/cancers14092337/s1, Supplementary Figure S1: OSF-2 (POSTN) is significantly up-regulated in TCGA HNSCC patient cohort (*n* = 566) comparing N vs PT; Figure S2: OSF-2 expression did not correlate with tumor localization in TCGA HNSCC patient cohort (*n* = 538); Figure S3: OSF-2 DNA methylation of six CpG sites in *n* = 528 HNSCC tumors and *n* = 82 normal tissue samples from the TCGA cohort; Figure S4: Osteopontin (SPP1) is significantly upregulated in HPV negative vs. positive tumors of the TCGA HNSCC patient cohort (*n* = 114); Figure S5: Spheroids of cisplatin-resistant pica cell line (PicaCis) exhibit significant chemoresistance towards different concentrations of cisplatin compared to wt cell line; Figure S6: Mutational analysis of OSF-2 gene using TCGA database (*n* = 493); Figure S7: Overexpression of OSF-2 in head and neck cancer cells did not alter tumor growth after subcutaneous injection into nude mice; Figure S8: OSF-2 secretion has no effect on cancer cell invasion. Figures S9–S13 Uncropped western blot figures. Supplementary Table S1: Clinical and histopathological characteristics of microarray cohort; Table S2: Clinical characteristics of TCGA Head and Neck Cancer (HNSC) cohort (*n* = 612); Table S3: Top 30 list of significantly up-regulated genes in PT vs. N (log2FC > 1.5, *p*-value < 0.0001); Table S4: List of used primers for RT-PCR, quantitative Real-Time PCR, and cloning; Table S5: Sequence of recombinant OSF-2 (BioVendor).
