# Supplementary material for: Impact of Secretion-Active Osteoblast-Specific Factor 2 in Promoting Progression and Metastasis of Head and Neck Cancer
Source: Cancers (Basel). 2022 May 9;14(9):2337. doi: 10.3390/cancers14092337 (PMC9106029; doi:10.3390/cancers14092337)
Supplement: Supplementary file 1 [file cancers-14-02337-s001.zip › Supplementary proof/Supplementary proof.pdf]

cancers

MDPI

Article

Impact of secretion-active osteoblast-specific factor 2 in promoting progression and metastasis of head and neck cancer

Désirée Gül[1,*], Andrea Schweitzer[1], Aya Khamis[1,2], Shirley K. Knauer[3], Guo-Bin Ding[4], Laura Freudelsperger[1], Ioannis Karampinis[5], Sebastian Strieth[6], Jan Hagemann[1], and Roland H. Stauber[1,4*]

Table S1 – Clinical and histopathological characteristics of microarray cohort

Patient	Primary site	Age	Sex	TNM	Grade	HPV
**1**	hypopharynx	47	f	T2 N2 M0	2	neg
**2**	hypopharynx	46	m	T4 N1 M0	2	neg
**3**	hypopharynx	48	m	T3 N1 M0	2	neg
**4**	hypopharynx	58	m	T3 N2 M0	2	neg
**5**	hypopharynx	72	m	T3 N2 M0	2	neg
**6**	hypopharynx	57	m	T1 N2 M0	3	neg
**7**	hypopharynx	57	m	T2 N3 M0	2	neg
**8**	hypopharynx	47	m	T2 N2 M0	2	neg
**9**	hypopharynx	56	m	T2 N3 M0	2	neg
**10**	larynx	68	m	T4 N2 M0	2	neg
**11**	larynx	56	m	T4 N3 M0	3	neg
**12**	oropharynx	58	f	T3 N2 M0	2	neg
**13**	oropharynx	56	m	T3 N2 M0	2	neg
**14**	oropharynx	49	m	T3 N1 M0	2	neg
**15**	oropharynx	53	m	T2 N2 M0	2	neg

cancers

MDPI

Table S2 – Clinical characteristics of TCGA Head and Neck Cancer (HNSC) cohort (n=612).

		n
Clinical T	T1	39
	T2	173
	T3	169
	T4a/b	203
Clinical N	N0	288
	N1	99
	N2a/b/c/-	180
	N3a/b/-	9
Clinical M	M0	571
	M1	5
Clinical UICC Stage [1]	Stage I	22
	Stage II	117
	Stage III	134
	Stage IV	311
Sex	Male	440
	Female	164

[1] 8th Edition

cancers MDPI

Table S3. Top 30 list of significantly up-regulated genes in PT*vs*.N ($log_2FC > 1.5$, p-value < 0.0001)

No.	Gene	Protein	log_2FC	p-value
1	**MMP1**	Matrix Metallopeptidase1	4,86	2,38E-09
2	**SPP1**	Osteopontin	3,27	2,01E-05
3	**POSTN**	Periostin, Osteoblast Specific Factor	3,06	9,40E-09
4	**COL1A1**	Collagen, Type I, Alpha 1	2,79	1,42E-06
5	**COL11A1**	Collagen, Type XI, Alpha 1	2,78	1,67E-05
6	**ASPN**	Asporin	2,73	6,72E-06
7	**INHBA**	Inhibin, Beta A	2,72	4,22E-07
8	**MMP3**	Matrix Metallopeptidase 3	2,71	2,00E-07
9	**COL5A2**	Collagen, Type V, Alpha 2	2,57	7,59E-07
10	**COL1A2**	Collagen, Type I, Alpha 2	2,56	1,22E-07
11	**MMP13**	Matrix Metallopeptidase 13	2,37	6,55E-07
12	**COL5A1**	Collagen, Type V, Alpha 1	2,23	5,68E-06
13	**CDH11**	Cadherin 11,Type 2 (OSF-4)	2,13	7,13E-05
14	**COL3A1**	Collagen, Type III, Alpha 1	2,11	1,19E-06
15	**LOX**	Lysyl Oxidase	1,98	2,32E-05
16	**LAMC2**	Laminin, Gamma 2	1,97	2,06E-05
17	**COL4A2**	Collagen, Type IV, Alpha 2	1,96	5,42E-08
18	**COL4A1**	Collagen, Type IV, Alpha 1	1,95	2,58E-08
19	**FN1**	Fibronectin 1	1,87	7,56E-06
20	**LOXL2**	Lysyl Oxidase-like 2	1,79	8,94E-08
21	**COL6A3**	Collagen, Type VI, Alpha 3	1,76	1,03E-06
22	**SULF1**	Sulfatase1	1,75	4,77E-05
23	**MAGEA4**	Melanoma Antigen Family A, 4	1,74	5,33E-06
24	**COL10A1**	Collagen, Type X, Alpha 1	1,71	3,33E-06
25	**SERPINE1**	Serpin Peptidase Inhibitor	1,70	9,23E-05
26	**FAP**	Fibroblast Activation Protein	1,67	9,01E-05

		Alpha		
**27**	**MFAP2**	Microfibrillar-Associated Protein 2	1,65	3,23E-07
**28**	**SPARC**	Osteonectin	1,64	3,77E-07
**29**	**IGFBP3**	Insulin-Like Growth Factor Binding Protein 3	1,64	6,06E-05
**30**	**PXDN**	Peroxidasin Homolog (Drosophila)	1,61	7,32E-06

Table S4. List of used primers for RT-PCR, quantitative Real-Time PCR, and cloning

**Primer**	**Sequence**
OSF-2 RT PCR for	5`-ATTAGGCTTGGCATCTGCTC-3`
OSF-2 RT PCR rev	5`-CTCGCGGAATATGTGAATCG-3
GAP-DH RT PCR for	5`-ACCACAGTCCATGCCATCAC-3`
GAP-DH RT PCR rev	5`-TCCACCACCCTGTTGCTGTA-3`
OSF-2 for	5`-AAACCGCGGATGATTCCCTTTTTACCCATGTT–3`
OSF-2 rev	5`-AAAGCTAGCCTGAGAACGACCTTCCCTTAATC–3`
OSF 2-ΔSec. for	5`-AAACCGCGGATGAACAATCATTATGACAAGATCTTG–3`
Secretion signal for	5`-CTAGCGGCGTTTATAGGGTTAACAATAAGCAGCAATAGTAG-3`
Secretion signal rev	5`-GGATGATTCCCTTTTTACCCATGTTTTCTCTACTATTGCTG-3`
RNA POL II for	5`-GCACCACGTCCAATGACAT-3`
RNA POL II rev	5`-GTGCGGCTGCTTCCATAA-3`

Table S5. Sequence of recombinant OSF-2 (BioVendor)

r-OSF-2 (Total 671 AA. MW: 75 kDa. UniProtKB Q15063. N-Terminal HisTag and Xa – cleavage site 23 AA)	MGHHHHHHHHHHSSGHIEGRHMRNNHYDKILAHSRIRGRDQGPNVCALQQILG<br>TKKKYFSTCKNWYKKSICGQKTTVLYECCPGYMRMEGMKGCPAVLPIDHVYGT<br>LGIVGATTTQRYSDASKLREEIEGKGSFTYFAPSNEAWDNLDSDIRRGLESNVN<br>VELLNALHSHMINKRMLTKDLKNGMIIPSMYNNLGLFINHYPNGVVTVNCARIIHG<br>NQIATNGVVHVIDRVLTQIGTSIQDFIEAEDDLSSFRAAAITSDILEALGRDGHFTL<br>FAPTNEAFEKLPRGVLERFMGDKVASEALMKYHILNTLQCSESIMGGAVFETLE<br>GNTIEIGCDGDSITVNGIKMVNKKDIVTNNGVIHLIDQVLIPDSAKQVIELAGKQQT<br>TFTDLVAQLGLASALRPDGEYTLLAPVNNAFSDDTLSMVQRLLKLILQNHILKVKV<br>GLNELYNGQILETIGGKQLRVFVYRTAVCIENSCMEKGSKQGRNGAIHIFREIIKP<br>AEKSLHEKLKQDKRFSTFLSLLEAADLKELLTQPGDWTLFVPTNDAFKGMTSEE<br>KEILIRDKNALQNIILYHLTPGVFIGKGFEPGVTNILKTTQGSKIFLKEVNDTLLVNE<br>LKSKESDIMTTNGVIHVVDKLLYPADTPVGNDQLLEILNKLIKYIQIKFVRGSTFKEI<br>PVTVY

cancers MDPI

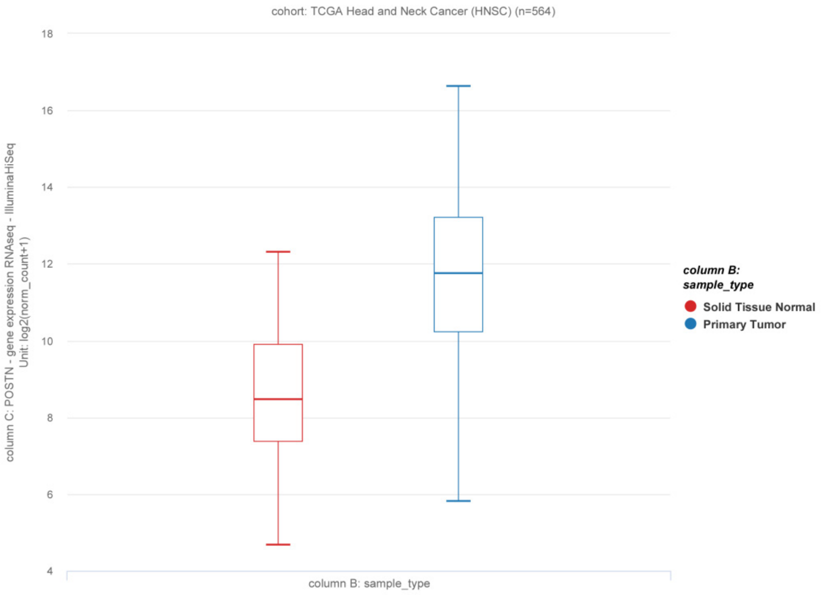

Figure S1. OSF-2 (*POSTN*) is significantly up-regulated in TCGA HNSCC patient cohort (n=566) comparing N vs PT. Welch's t-test p = 2.341e-16

cancers MDPI

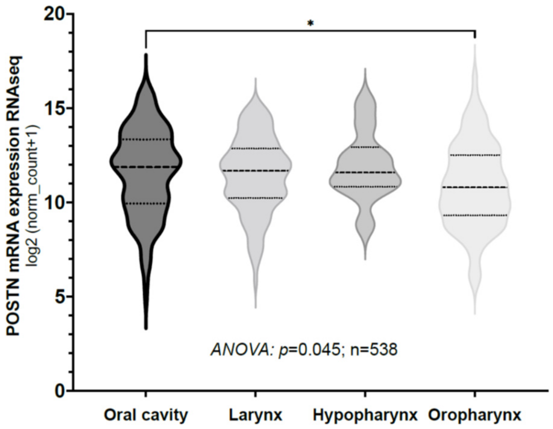

Figure S2. OSF-2 expression did not correlate with tumor localization in TCGA HNSCC patient cohort (n=538).

cancers

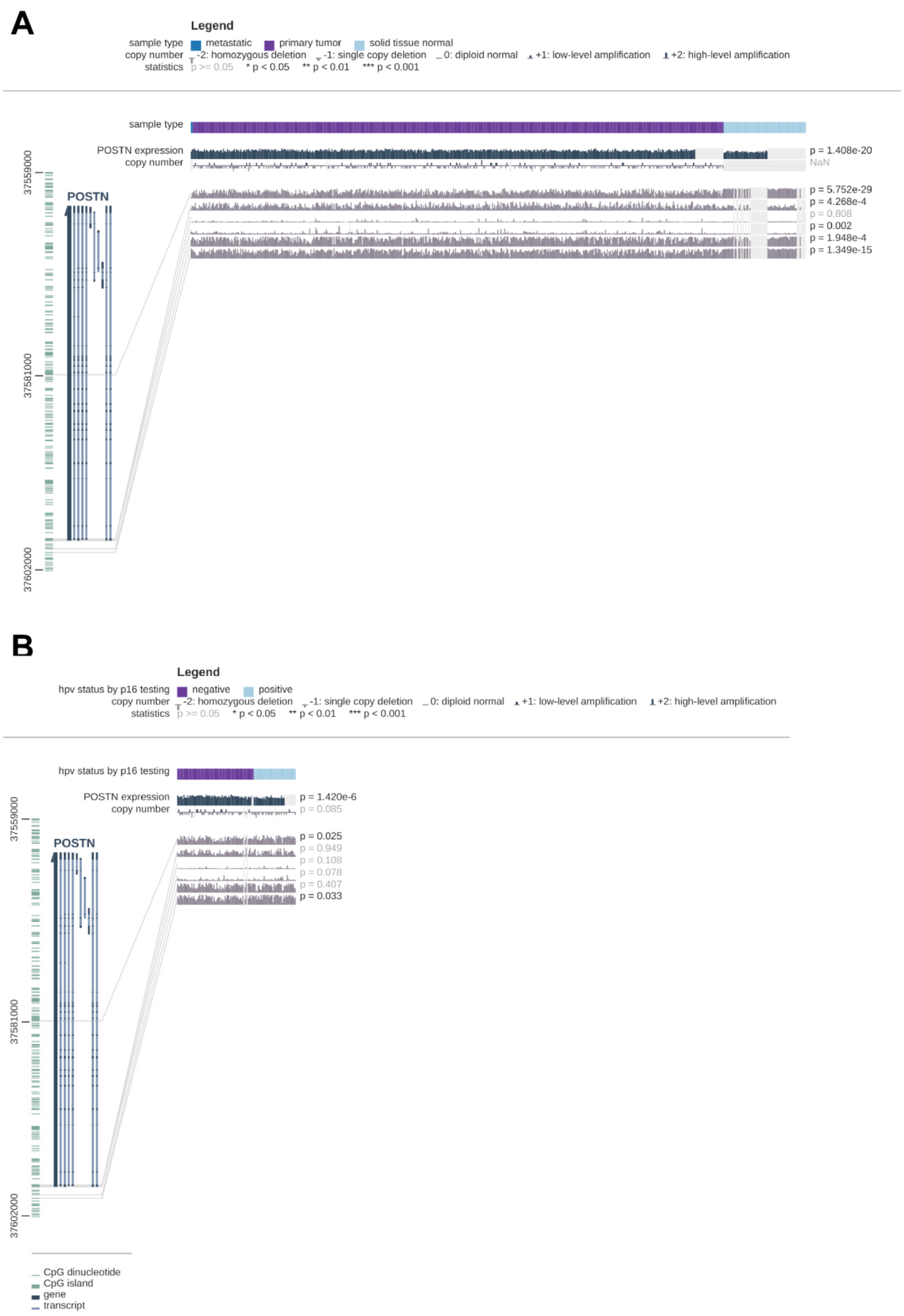

Figure S3. ***OSF-2/POSTN*** **DNA methylation of six CpG sites in n=528 HNSCC tumors and n=82 normal tissue samples from the TCGA cohort** (Infinium HumanMethylation450 BeadChip; cg23202139, cg04922971, cg24719107, cg05604486, cg13634560, cg18341491). Analysis was performed with MEXPRESS online tool (https://www.mexpress.be) (A) Methylation in 5 of 6 CpG sites is significantly decreased in PT vs. N correlating with increased POSTN expression levels. (B) POSTN methylation is reduced in HPV negative tumors compared to HPV positive samples. p values as indicated.

cancers

MDPI

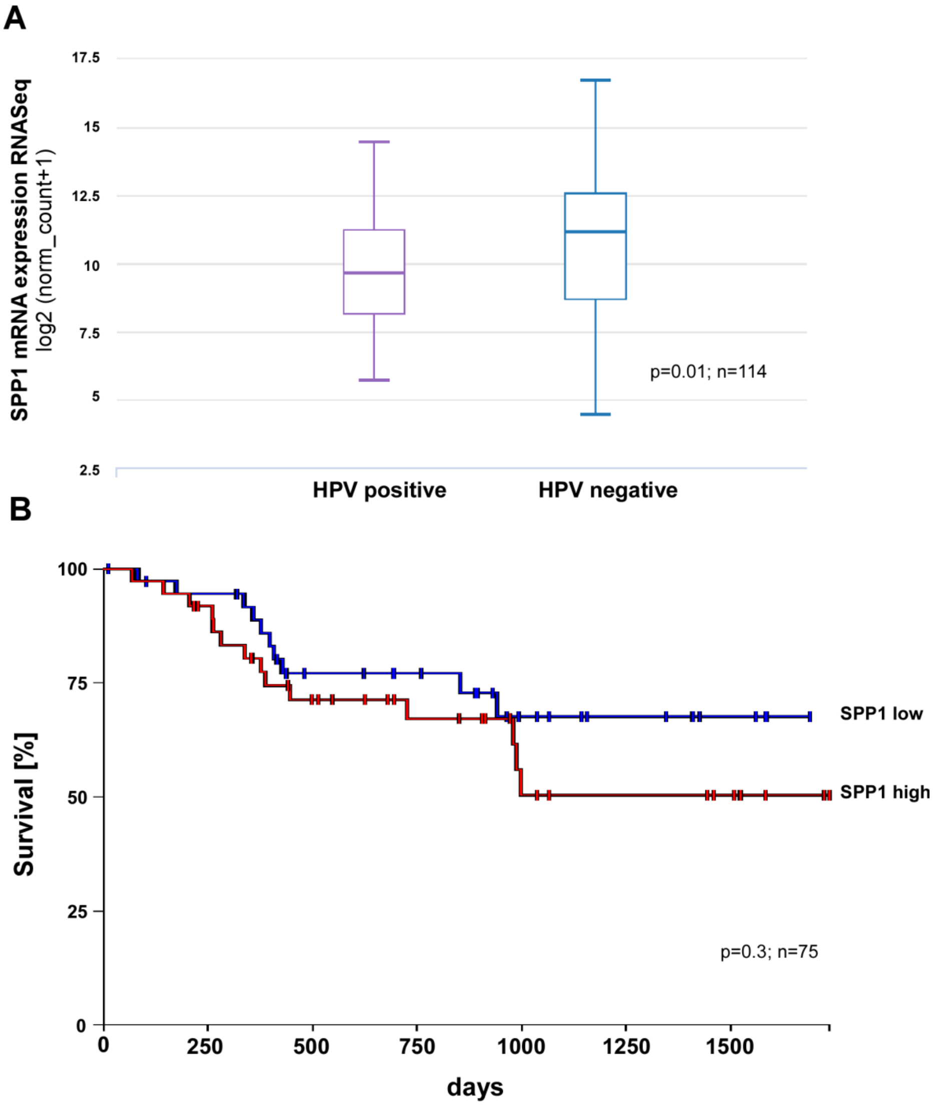

Figure S4. (A) Osteopontin (*SPP1*) is significantly upregulated in HPV negative vs. positive tumors of the TCGA HNSCC patient cohort (n=114). (B) High *SPP1* expression levels did not correlate with overall survival of HPV negative HNSCC patients. n=75; p=0.3.

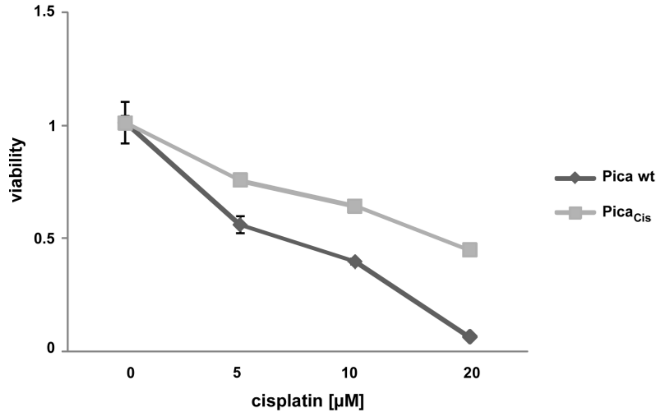

Figure S5. Spheroids of cisplatin-resistant pica cell line (PicaCis) exhibit significant chemoresistance towards different concentrations of cisplatin compared to wt cell line.

cancers MDPI

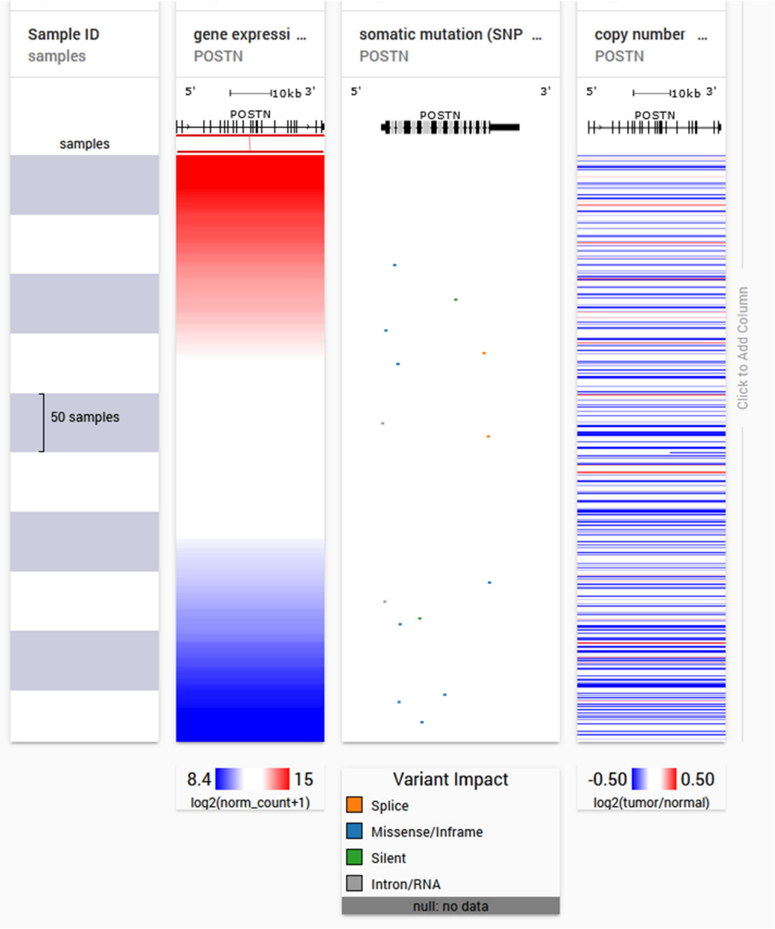

Figure S6. Mutational analysis of *OSF-2* gene using TCGA database (n=493). Different types of mutations (middle column) were found in 15 HNC patients. There seems to be no correlation between high OSF-2 expression (left, red) and the occurrence of somatic mutations and/or copy number variations (right).

cancers

MDPI

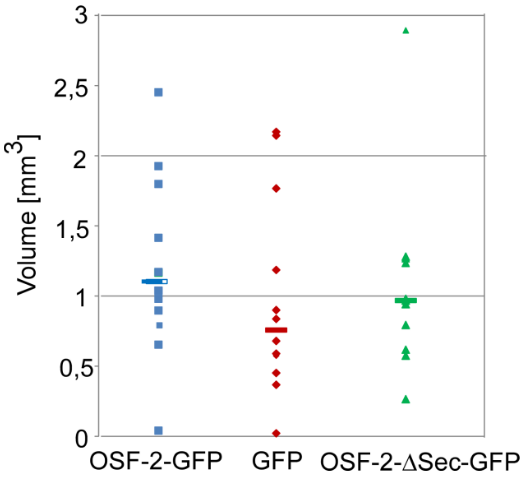

Figure S7. Overexpression of OSF-2 in head and neck cancer cells did not alter tumor growth after subcutaneous injection into nude mice. The average ratio of the tumor volume between tumors derived from periostin-overexpressing cells and control cells is less than 2-fold.

cancers MDPI

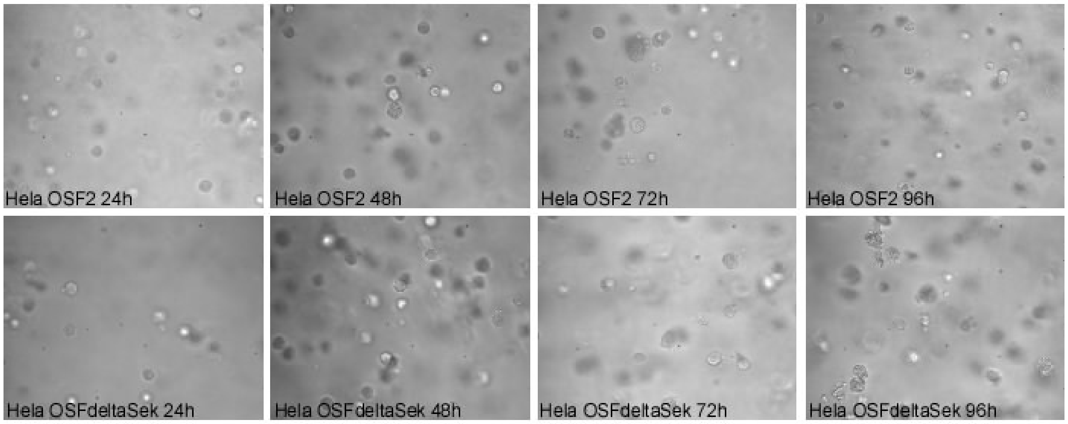

Figure S8. OSF-2 secretion has no effect on cancer cell invasion. Matrigel invasion assay of transfected HeLa cells expressing secretion active *versus* secretion deficient OSF-2 (deltaSek). 50 - 100 µl diluted matrigel (BD Bioscience) was pipetted into the upper chamber of the 24-well transwell (Falcon BD, 8 µm pore size). 24h after transfection, $1x10^5$ cells were seeded in 100 µl of cell suspension in medium with 1% FCS into upper chamber of transwell. After gelling at 37° C for at least 4-5 hours, lower chamber was filled with 10 % FCS medium, and incubated at 37° C. Cells were observed for 4 days.

cancers

MDPI

Raw Data

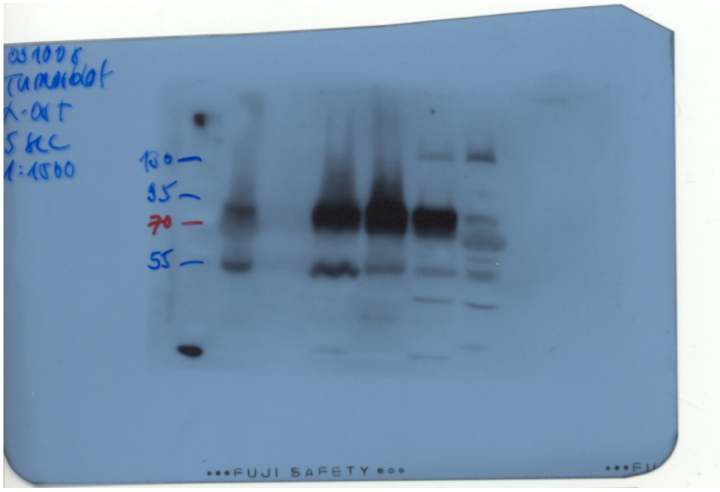

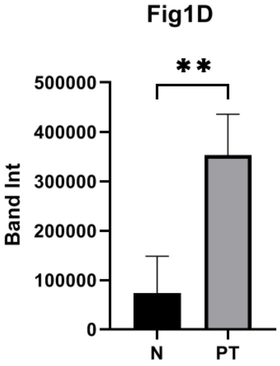

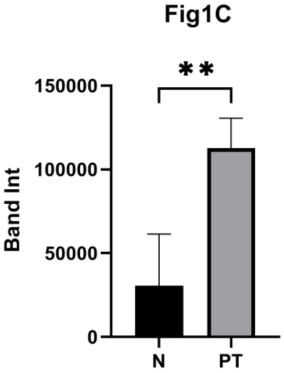

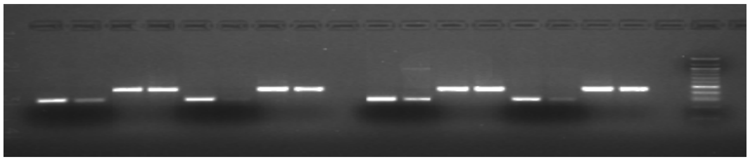

Figure S9. Western blot (upper) and agarose gel (lower) referring to validation of microarray data (Figure 1C, D) including densitometric analysis of bands (n=2) (right).

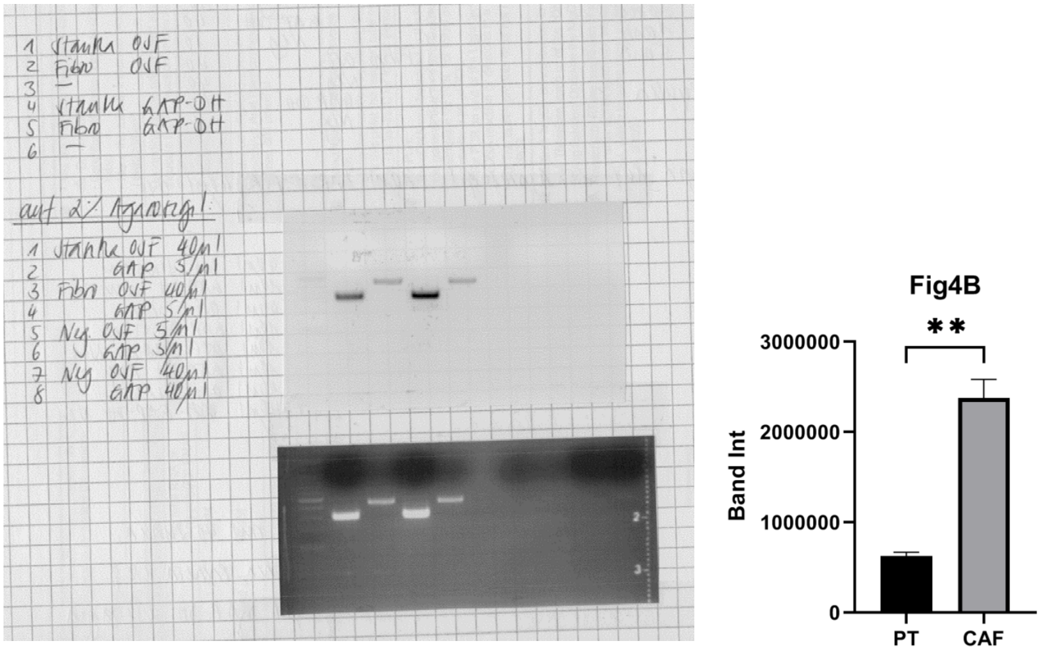

Figure S10. Agarose gel referring to RT-PCR analysis of POSTN expression in tumor cells and CAFs (Figure 4B) including densitometric analysis of bands (n=2) (right).

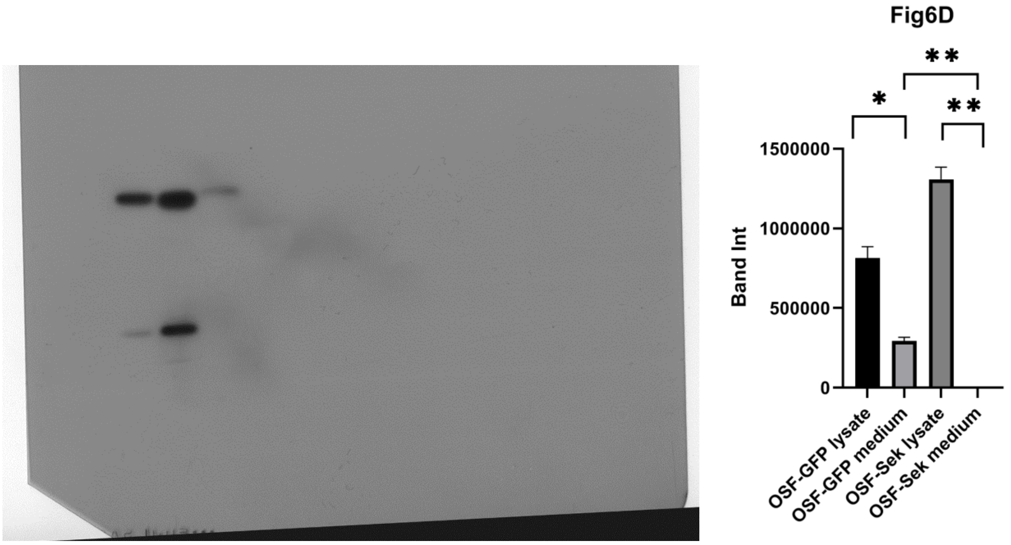

Figure S11. Western blot referring to analysis of OSF-2 secretion signal (Figure 6D) including densitometric analysis of bands (n=2) (right).

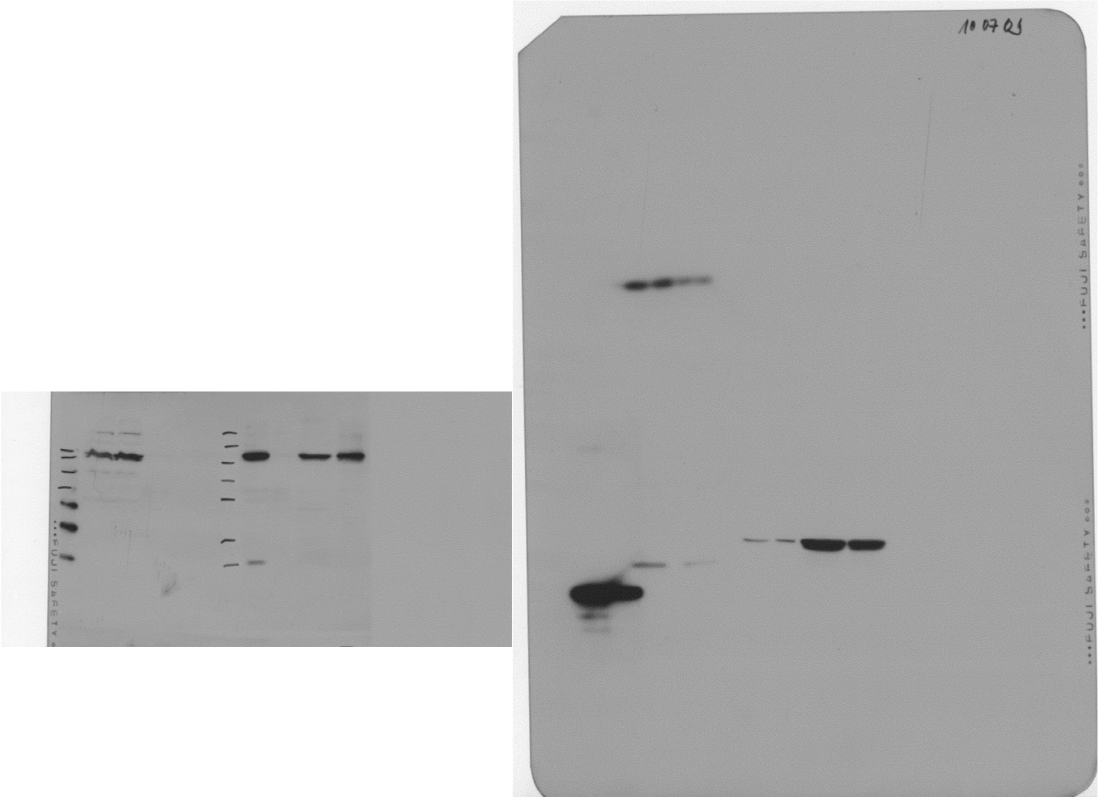

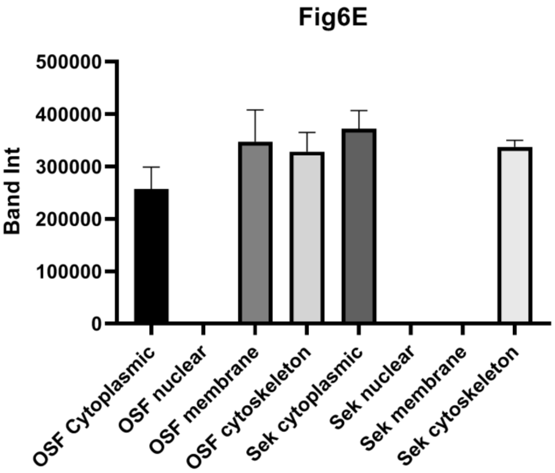

Figure S12. Western blots referring to cell fractionation analysis of secreted/ non-secreted OSF-2 (Figure 6E). a-OSF Ab (left), loading controls (right), and densitometric analysis of bands (n=2) (below).

cancers

MDPI

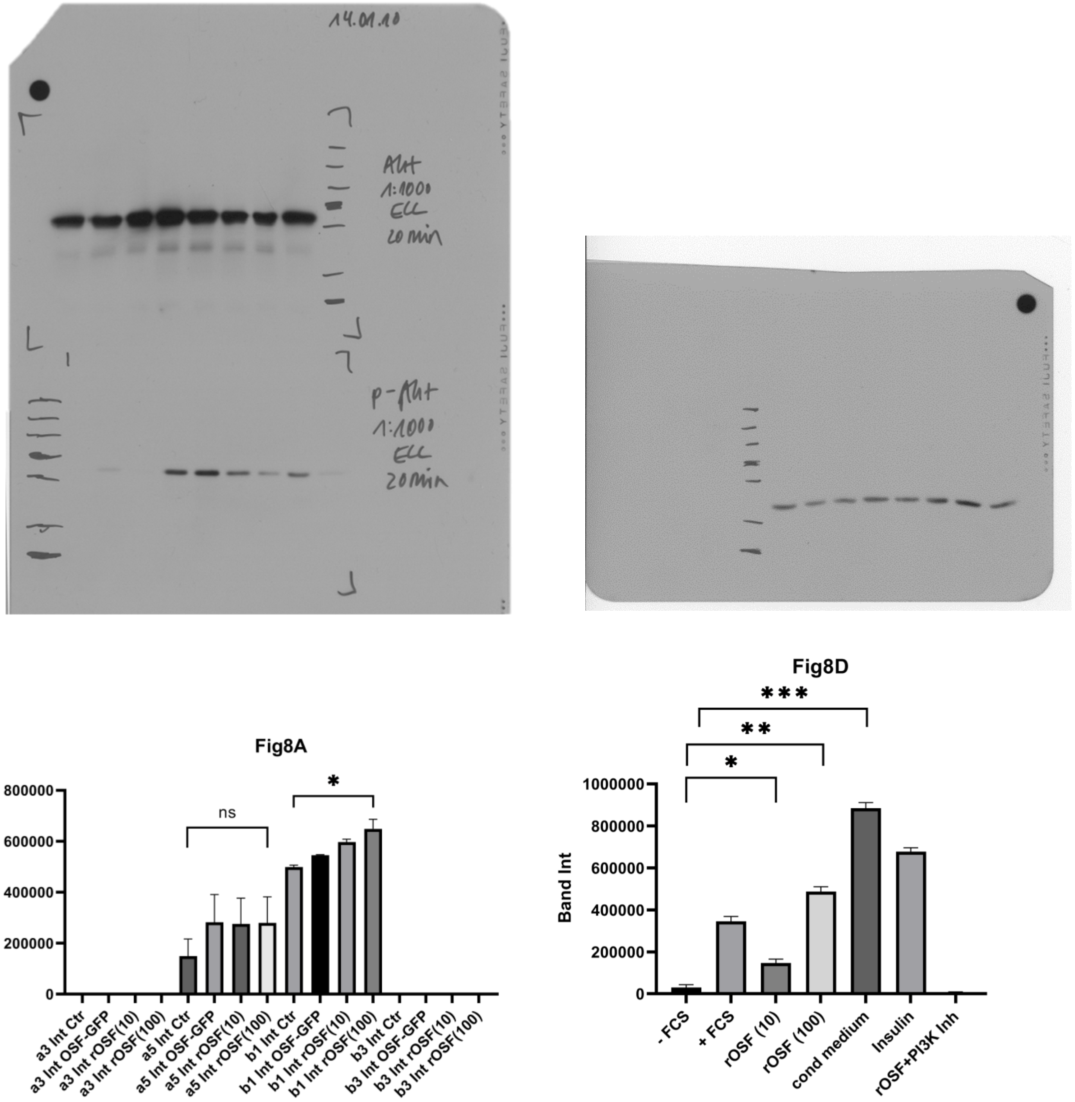

Figure S13. Western blots and densitometric analyses referring to Figure 8 A and D. a-pAkt Ab (left, lower), a-Actin Ab (right). Densitometric analysis of bands (n=2) below.
